# Time-resolved probing of laser-induced nanostructuring processes in liquids

**DOI:** 10.3762/bjnano.16.74

**Published:** 2025-07-02

**Authors:** Maximilian Spellauge, David Redka, Mianzhen Mo, Changyong Song, Heinz Paul Huber, Anton Plech

**Affiliations:** 1 Laser Center Hochschule Munich, Munich University of Applied Sciences, Lothstr. 34, 80335 Munich, Germany; 2 Technical Chemistry I and Center for Nanointegration Duisburg-Essen (CENIDE), University of Duisburg-Essen, 45141 Essen, Germany; 3 New Technologies Research Center, University of West Bohemia, Plzen CZ-30100, Czech Republic; 4 SLAC National Accelerator Laboratory, Menlo Park, 94025, CA, USA; 5 Department of Physics, POSTECH, Pohang 37673, Korea; 6 Institute for Photon Science and Synchrotron Radiation, Karlsruhe Institute of Technology (KIT), Postfach 3640, D-76021 Karlsruhe, Germany

**Keywords:** electron diffraction, laser processing in liquids, optical imaging, optical spectroscopy, pump–probe, single objects, time-resolved probing, X-ray scattering

## Abstract

Laser synthesis and processing of colloids (LSPC) in liquids has gained widespread applications in producing nanomaterials of different classes of solids. While the technical processes in different cases of ablation, fragmentation or colloidal fusion may look macroscopically different in each application, the underlying fundamental mechanisms are always the same cascade of laser interaction with matter, non-thermal or thermal energy deposition, phase transitions, and the subsequent structure formation processes. Disentangling these mechanisms represents a veritable challenge, as ultrafast and structurally sensitive experimental methods are required. This review presents a discussion of how state-of-the-art experimental protocols using ultrafast lasers and sensitive structural probes, such as electrons or X-rays are able to address this challenge. In particular, it is possible to investigate LSPC on single objects using single probe pulses and avoid accumulation effects in a heterogeneous sample. The presented results capture structure formation with femtosecond and atomic scale resolution. Ultrafast time-resolved probing approaches are key to revealing the transient states and pathways that govern material transformation in LSPC.

## Review

### Introduction

Laser synthesis and processing of materials with emphasis on structure formation on the nanoscale offers a multitude of pathways to desired structure-related functions [1]. In green processes, nanostructures and nanoparticles (NPs) can be produced that serve applications in many fields, such as heterogeneous catalysis [2–3], (bio)photonics [4], and coating or device fabrication [5]. In a bottom-up approach the products of the laser-based synthesis are not defined by direct spatial control, but by harnessing the energetic and structural relaxation pathways of fundamental reactions that span from atomic to macroscopic length scales. Consequently, it is paramount to understand the complex cascades that follow laser irradiation of condensed matter. Of particular importance here is the processing in the presence of liquids surrounding the irradiated material, but also exchanging energy and material with the excited loci.

LSPC commonly encompasses laser ablation in liquid (LAL), which allows for producing NPs from a surface of virtually any solid [6–9], laser fragmentation in liquid (LFL) to further reduce dimensions of particles down to few-atom clusters [10–13], as well as laser fusion or laser melting in liquid (LML) [14–15]. The latter is used to achieve the opposite effect of increasing particle size with the aim for high quality in shape or size.

The presence of a liquid in laser processing, on the one hand, has practical advantages, such as simple handling and safe suspension of the products for further use. On the other hand, the interaction of the irradiated surfaces and NPs with the liquid forms an active interface for energy exchange, leading to extreme cooling rates of 1000 K per nanosecond, which quenches melted particles, generates ablation and fragmentation products with a high defect density, and enhances catalytic activity. In addition, the liquid may also participate in the reaction by electrostatic stabilization [12], formation of gases [16], or chemical interaction with the target to enhance redox reactions or passivating coatings [17–19].

The understanding of LSPC in liquids has been largely stimulated by theoretical approaches and simulations. In a thermodynamic approach, the equilibration of excited electrons with the phonon bath is described in the two-temperature model (TTM) [20–22], which conveys important consequences, such as providing timescales for lattice heating or explaining nonlinearities in excitation through electron heat capacity [23]. One notable exception from the universality of the TTM that limits its generalization to more degrees of freedom is the impact of non-thermalized electrons on structure formation via direct and near-field forces [24–27]. Furthermore, the application of TTM is hampered by the relatively high uncertainty of material parameters such as the temperature- or field-dependent interaction cross section or the non-linear heat capacities [28–31]. Heat flow and phase transitions can be well modeled by classical approaches down to the nanometer scale in a local thermodynamic equilibrium [32–34]. A synthesis of the different coevolving phenomena with excitation and dissipation requires more detailed numerical approaches [35] or simulations. Molecular dynamics (MD) simulations [36–37] are widely used to predict phase transitions or structure formation on the nanoscale [38–39], but ultimately also require interfacing the simulations with other models, such as TTM [40–41] or large-scale hydrodynamics [42–45]. This integration approach leads to computationally demanding studies that eventually show convergence with experimental results.

**Figure 1:**
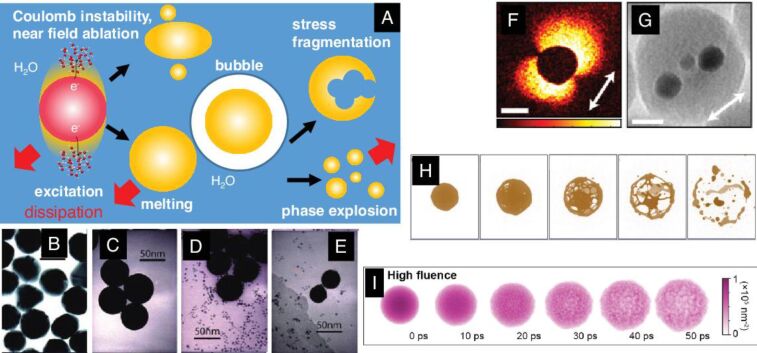
Scheme of fundamental structural relaxations of laser-excited metal NPs in liquid environment. (A) After short-pulse excitation, the laser field imposes anisotropic forces due to surface plasmon resonance (SPR), which can lead to a Coulomb instability with spallation or near-field ablation. Particles melt after high-intensity excitation, which is accompanied by bubble formation in the liquid after heat dissipation. Possible channels for fragmentation include strain-induced explosion as well as thermal phase explosion. (B–E) Several stages of morphology changes of excited gold particles after single-pulse irradiation at 20, 50, 100, and 200 J/m^2^ (from left). (F) Photon-induced near-field imaging of the SPR of gold particles in a silica shell in an electron microscope together with the directional fission due to the directed charge emission (G). (H) results of numerical simulations of femtosecond particle excitation (15 nm) at 1000 J/m^2^ in a delay sequence from 46 to 281 ps (from left). (I) Numerical simulation of femtosecond particle excitation (20 nm) at an absorbed fluence of 84 J/m^2^ in a delay sequence up to 50 ps. Figure 1B–E was adapted with permission from [51], Copyright 2011 American Chemical Society. This content is not subject to CC BY 4.0. Figure 1F and Figure 1G were reprinted with permission from [52], Copyright 2019 American Chemical Society. This content is not subject to CC BY 4.0. Figure 1H was adapted with permission from [39], Copyright 2015 American Chemical Society. This content is not subject to CC BY 4.0. Figure 1I was reproduced from [53] (© 2024 J. Hwang et al., some rights reserved; exclusive licensee American Association for the Advancement of Science, distributed under the terms of the Creative Commons Attribution-NonCommercial 4.0 International License, https://creativecommons.org/licenses/by-nc/4.0/). This content is not subject to CC BY 4.0.

Generally, the classification of the mechanisms in LSPC identifies three main pathways that are pictured schematically in Figure 1: (i) thermal processes, where the condensed matter is heated to high lattice temperatures, leading to melting, reshaping (Figure 1B,C), evaporation, and phase explosion near the critical point (Figure 1H) [39,46–48]; (ii) stress-induced decompositions, where competition between heating and expansion leads to spallation or cavitation [36,49–50] (Figure 1I); (iii) non-thermal processes, where excessive electron emission leads to material decomposition via charge forces [51–52] (Figure 1D–G). Discriminating between these mechanisms in experiments is challenging and requires carefully designed approaches that resolve the processes spatially, temporally, and energetically.

This review introduces specific questions and results pertaining to the understanding of laser material processing relevant to NP synthesis and manipulation. Since a manifold of individual mechanistic processes can be initiated by intense pulsed laser excitation, it is challenging to disentangle the structural relaxations crossing all the time and length scales, as well as to discriminate individual structural pathways from an average sampling over repetitive pulses, varying fluence or ensemble of nonidentical NPs and structures. While optical methods have been paramount to understanding structural processes and verifying proposed pathways, we describe spatially and temporally resolved optical reflectivity and short-wavelength scattering methods that are able to directly reveal structural motifs. Ultimately, it is possible to investigate single NPs with a single shot before complete destruction. With these methods, we describe structural pathways for particle generation, fragmentation, and energy coupling to the liquid environment to establish a mechanistic knowledge of fundamental and applied processes.

The first chapter entitled “Ultrafast light–matter interaction directly observed via single-pulse, single-particle imaging” describes the processes initiated by laser-excitation of an individual NP probed by X-ray scattering up to delay times of a few tens of picoseconds. The second chapter “Structural dynamics in liquids” reports on the application of ultrafast time-resolved electron scattering to investigate early-time structural relaxation and chemical bond dynamics in laser-induced ionization of liquid water. In the third chapter “Nanoparticle excitation in an ensemble and energy exchange with medium”, an approach is shown on how reactions of both the excited NPs as well as the interaction with the surrounding liquid can be resolved in one experiment. And finally, a fourth chapter provides a state-of-the-art overview on the subject “Large-scale optical probing of laser ablation dynamics in liquids”. Here, the relaxation dynamics of a few tens of square micrometer large laser-excited surface, as used in many setups designed for NP generation by laser ablation in liquid, is probed with optical methods from the picosecond time domain up to the final state in the millisecond domain.

### Ultrafast light–matter interaction directly observed via single-pulse, single-particle imaging

The interaction of intense laser fields with matter has significantly enriched our understanding of material deformation and phase transition kinetics, drawing increasing attention to strongly driven physical systems [54]. Hidden material phases revealed under non-equilibrium conditions have expanded research on photoinduced ultrafast phenomena, enabling precise control of material properties [55–58]. This advancement has catalyzed new directions in nanomaterial processing and synthesis, such as producing controlled NPs through ultrafast laser–matter interactions. A notable example is the production of monodisperse NPs in aqueous solutions via laser pulse exposures [1,59], demonstrating practical applications and underscoring the need for deeper insights into non-equilibrium light–matter interactions.

**Figure 2:**
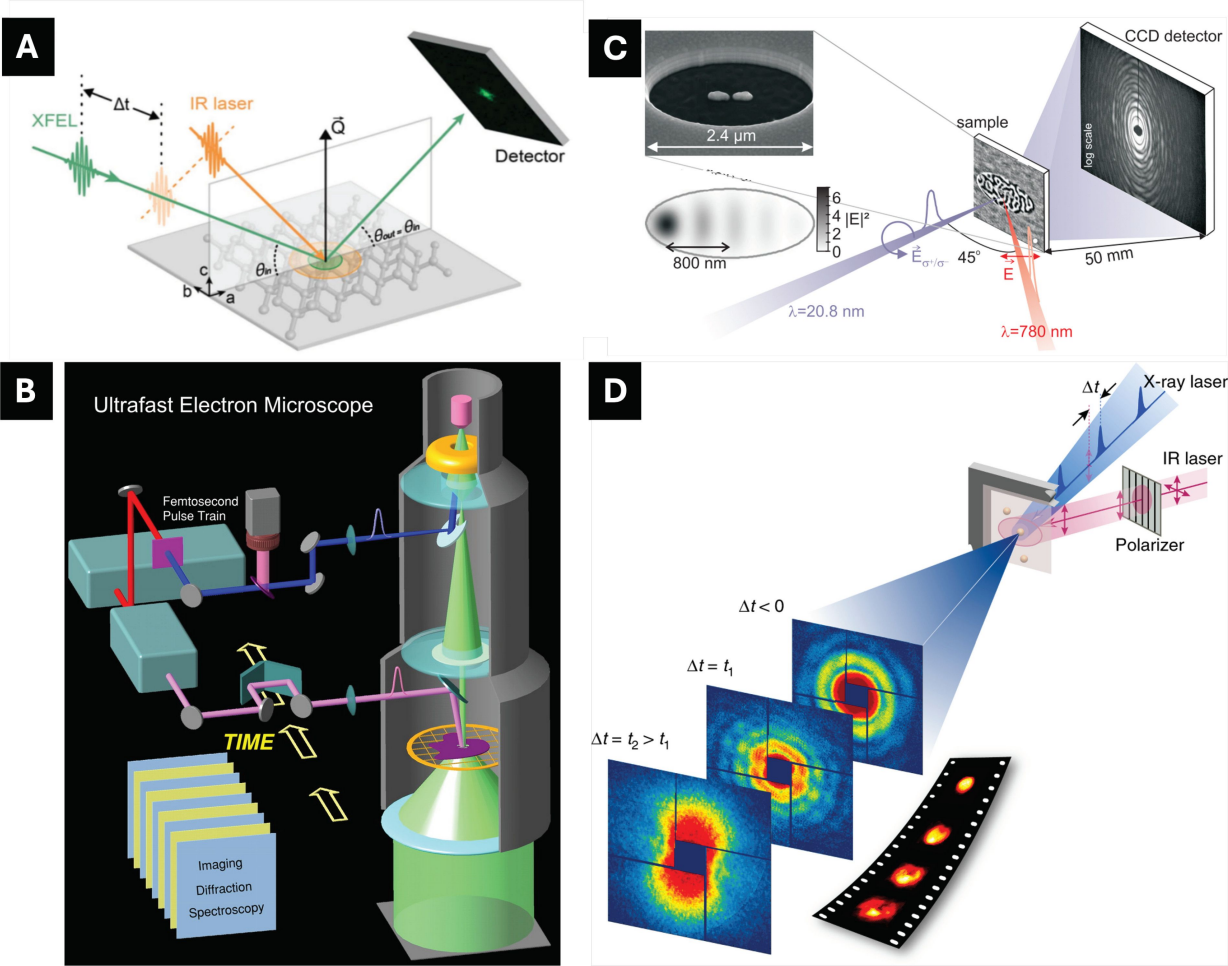
Time-resolved experiments using ultrashort X-ray or electron pulses via a pump–probe method. (A) Time-resolved X-ray diffraction investigates the ultrafast dynamics of various long range orders in crystals. (B) Lab-based four-dimensional transmission electron microscope for direct imaging of single specimens or crystalline lattices. (C) Ultrafast holographic X-ray imaging investigation relaxation systems. (D) Single particle dynamics imaging with single XFEL pulses in directly imaging the irreversible kinetics. Figure 2A was reproduced from [66] (© 2023 H. Lee et al., published by IUCr Journals, distributed under the terms of the Creative Commons Attribution 4.0 International License, https://creativecommons.org/licenses/by/4.0). Figure 2B was reprinted from [67], Copyright (2005) National Academy of Sciences, U.S.A. This content is not subject to CC BY 4.0. Figure 2C was reprinted with permission from [64], Copyright (2014) by the American Physical Society. This content is not subject to CC BY 4.0. Figure 2D was reproduced from [68] (© 2019 Y. Ihm et al., published by Springer Nature, distributed under the terms of the Creative Commons Attribution 4.0 International License, https://creativecommons.org/licenses/by/4.0).

Experimental approaches to uncover the underlying reaction kinetics of such complex interactions have been diverse and continuously evolving [26,60–64]. X-ray or electron diffraction techniques (Figure 2), implemented via pump–probe schemes and combined with femtosecond ultrashort pulses, have enabled the observation of ultrafast atomic-scale motions [65]. During these transitions, transient and previously unknown material phases emerge, highlighting the existence of states accessible only under strongly driven non-equilibrium conditions. This insight has spurred further investigations into ultrafast reaction kinetics beyond the thermodynamic limit. However, these diffraction techniques often yield ensemble-averaged dynamics with angstrom-scale spatial resolution, as they rely on well-resolved interference fringes from periodic crystalline structures. Consequently, local structural variations are frequently obscured and data interpretation is constrained by predefined models. These limitations become even more pronounced when studying non-equilibrium states, such as laser-induced material deformation kinetics, where established knowledge is still developing.

To address these challenges, direct imaging probes with high spatial and temporal resolution have been developed [68–70]. Direct imaging offers the advantage of studying non-equilibrium phase transitions without relying on a priori models.

In pump–probe imaging experiments, specimens are excited using femtosecond optical laser pulses and subsequently imaged at defined time delays to capture their dynamic responses. This method is particularly advantageous for visualizing structural changes in single particles. Although technical challenges remain in achieving sufficient signal-to-noise ratio, single-particle imaging uniquely resolves local fluctuations that are otherwise averaged out in ensemble measurements. Recognizing that structural inhomogeneities drive emergent material properties, the ability to observe such local fluctuations is increasingly vital. Single-particle imaging with several nanometers spatial and several tens of nanoseconds temporal resolution has been demonstrated using electron microscopy [71]. By integrating a photocathode-based ultrashort electron pulse generator into a lab-based electron microscope, dynamic imaging of single specimens via pump–probe electron microscopy became possible. For instance, a spin state transition in the metal–organic framework Fe(pyrazine)Pt(CN)_4_ NPs was induced by nanosecond laser pulses, resulting in a high- to low-spin transition accompanied by morphological deformations coupled with lattice dynamics.

Femtosecond X-ray laser pulses from X-ray free-electron lasers (XFELs) have further revolutionized dynamic X-ray imaging. Coherent diffraction imaging (CDI) [68–69,72–73] utilizes the spatial coherence of X-ray laser pulses such that the fully coherent diffraction pattern of an object is reconstructed by iterative phase retrieval algorithms retrieving the object density. This technique is particularly suited for time-resolved imaging of dynamic processes, which show transient states that exist only for femtoseconds. While the initial demonstration used the forward scattered intensity (small-angle X-ray scattering, SAXS) to derive the spatial scattering length distribution (related to material density), an extension enabled reconstructing scattering at Bragg peaks (in wide-angle X-ray scattering (WAXS) geometry) to specifically reconstruct the distribution of crystallinity and lattice strain [74]. In pump–probe configuration, photoinduced strain field oscillations in single Au nanocrystals [75] have been resolved. These studies revealed large-scale NP deformations caused by thermally excited elastic strains. However, stroboscopic measurements like these are limited to systems that recover their original states after perturbation and are unsuitable for studying irreversible kinetic reactions in strongly driven non-equilibrium phenomena.

The development of single-pulse, single-particle imaging using XFELs has enabled nanoscale-resolution observations of ultrafast irreversible processes. XFELs’ unique combination of spatial coherence, ultrahigh brightness, and femtosecond pulse durations provides a powerful probe for studying interactions between strong light fields and matter. Investigations into photoinduced melting, a well-known, yet incompletely understood phenomenon, have highlighted the role of non-thermal processes [29,76–78]. For example, time-resolved resonant X-ray scattering studies have directly observed the ultrafast reconfiguration of bonding orbitals in Ge crystals, revealing a coexistence of thermal and non-thermal melting mechanisms [66,79]. Local regions breaking translational symmetry and initiating the melting of the entire specimen were revealed through single-pulse, single-particle imaging, providing a microscopic understanding of the melting transition [68].

The single-particle dynamic CDI discussed here can be compared with the aforementioned Bragg CDI, highlighting their distinct technical advantages for addressing specific scientific challenges. Bragg CDI measures coherent diffuse scattering patterns around a specific Bragg reflection of a specimen. A full three-dimensional mapping of the coherent scattering pattern along a particular Bragg reflection is achieved by rocking the specimen while maintaining the Bragg reflection condition. The oversampled coherent 3D diffraction pattern obtained is then phase-retrieved to image the specimen, revealing strained lattice deformations along the direction parallel to the Bragg reflection. Whilst this Bragg CDI offers exceptional sensitivity to lattice strain fields formed over the whole specimen, its imaging capability relies on successfully collecting a complete 3D map of the diffuse pattern surrounding a specific Bragg reflection. This method requires initial alignment of the specimen to a Bragg reflection and repeated restorations to its original, intact state during the collection of the entire 3D map via crystal rocking. Consequently, it is not well suited for studying nonequilibrium kinetics such as strong light–matter interactions that involve irreversible phase changes.

In contrast, the single-particle, single-pulse CDI introduced here overcomes such limitations. Each single-pulse coherent diffraction pattern is acquired in transmission geometry using a single X-ray pulse, eliminating the need for specimen alignment to a specific Bragg angle. This ensures that the specimen can be completely damaged after a single-pulse measurement, as fresh specimens can be used for subsequent single-pulse exposures. The only requirement is that all specimens must be structurally (morphologically) identical to the extent dictated by the image resolution. This condition is practically achievable due to remarkable advancements in monodisperse NP synthesis. Each single-pulse coherent diffraction pattern is collected from a new, fresh specimen with completely independent experimental parameters. Time-resolved imaging is accomplished by controlling the delay time between the pump and the probe pulses. The reliability of experimental data is ensured by collecting multiple datasets for a given delay time, thereby avoiding misinterpretations of reaction kinetics caused by fluctuations in individual single-particle images.

Single Au nanospheres were irradiated with femtosecond infrared laser pulses, rapidly increasing the electron temperature to several tens of thousands of kelvins within a few hundred femtoseconds. Photoexcited hot electrons in metallic Au transferred their excess kinetic energy by colliding with other conduction electrons and Au ions, which subsequently increased the lattice temperature, reaching equilibrium within a few tens of picoseconds.

Interestingly, ultrafast single-particle imaging revealed unexpected phenomena that deviate from the conventional TTM that describes energy transfer between electrons and the lattice. Although the dynamics of photoexcited hot electrons were expected to uniformly affect the entire specimen within a few picoseconds, the experiment instead observed inhomogeneous and laser-polarization-dependent anisotropic melting. This indicates that residual effects from the initial electron dynamics play a dominant role in influencing ionic dynamics at later stages, persisting for several to tens of picoseconds.

Localized surface plasmon formation in metallic NPs, followed by their annihilation, have been identified as key contributors to the observed anisotropic melting behavior. These findings, corroborated by MD simulations that incorporate the TTM (TTM-MD), suggest that energy accumulation in localized hotspots drives anisotropic melting. Nevertheless, direct experimental evidence linking electronic and ionic dynamics across multiscale time domains remains elusive.

**Figure 3:**
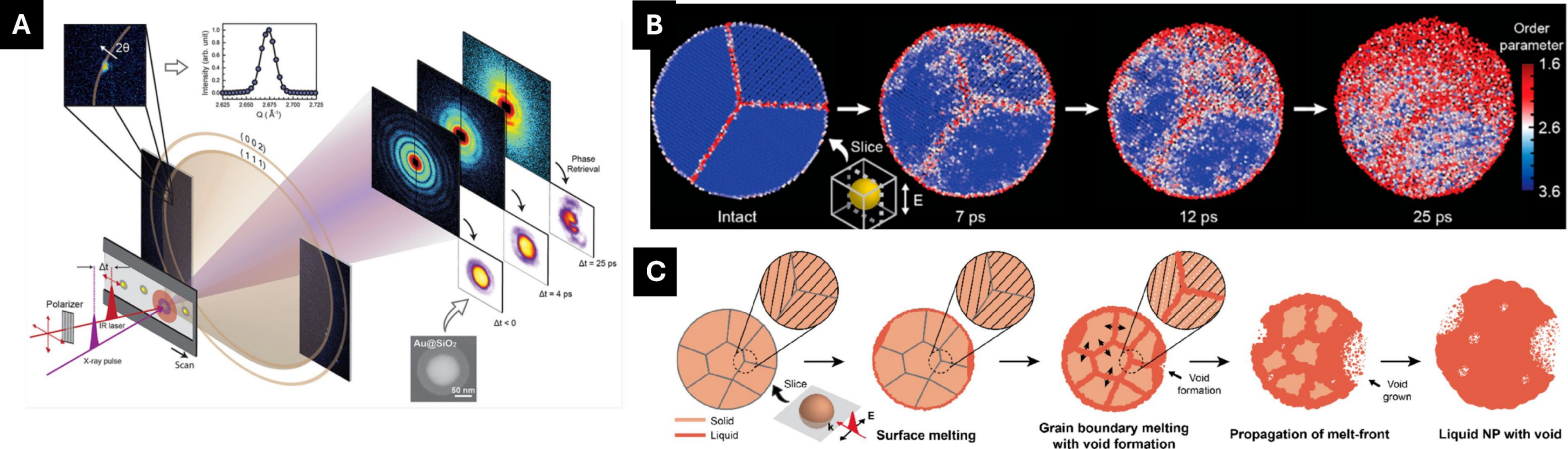
Single-particle investigation via multiplexing experiments. (A) An XFEL single-pulse multiplexing experiment. By implementing a wide-angle detector close to the interaction spot, the crystal structure is monitored in addition to the sample densities in the projection images. (B) TTM-MD, by corroborating the atomic structure information and heterogeneous sample morphology, describes the melting process in atomic scale. (C) Overall photoinduced melting process of single Au NP. Figure 3 was adapted with permission from [80], Copyright 2023 American Chemical Society. This content is not subject to CC BY 4.0.

Multiplexing probes, such as the combination of imaging and crystal diffraction, have advanced the understanding of complex phenomena by narrowing the gap between theory and observation (Figure 3) [80–81]. In an experiment combining single-particle imaging with wide-angle crystal diffraction, additional detectors near the specimen captured crystal Bragg reflections, offering new insights into photoinduced melting. The study revealed that local atomic disorder begins at the surface before the overall lattice expands, with the melt front progressing from the particle surface and grain boundaries, as evidenced by a reduction in crystal domain sizes. Direct imaging of void formation further deepened the understanding of the process, showing that voids act as primary drivers of the transition, corroborated by TTM-MD simulations and additional single-particle experiments. These voids form due to local pressure differences caused by energy accumulation in hot spots via photoexcited localized surface plasmons, with the overall melting and particle disintegration resembling an inverted crystal nucleation process, where voids act as seeds and their expansion mirrors crystal growth [53].

While a fundamental understanding of phase transitions driven by ultrafast and intense laser fields is emerging, many details remain elusive. A key unresolved issue is how femtosecond-scale photoinduced electron dynamics can drive much slower ionic motion with several orders of magnitude timing lag, a phenomenon that demands paradigm-shifting insights supported by direct experimental evidence, unconstrained by traditional equilibrium thermodynamics. Alongside the ongoing effort to understand ultrafast laser–matter interactions, focused research on manipulating phase-transition kinetics using femtosecond laser pulses is critical. Such research promises to improve the ability to engineer functional materials through on-demand customization.

Recognizing that photoexcited electrons serve as the initial point, it is intuitive to envision controlling localized surface plasmons through the wavelength and polarization state of femtosecond laser pulses [82]. Active research in this domain is anticipated to facilitate the precise optical control of material phases. Furthermore, advances in imaging technologies, particularly those enabling ultrafast, three-dimensional, and atomic-scale time-resolved imaging, will be pivotal in elucidating local atomic-scale structural dynamics directly associated with photoinduced electron behavior. The introduction of megahertz repetition rate XFELs is expected to significantly propel these endeavors. This will coincide with the substantial production of single-pulse data, which awaits prompt online analysis to facilitate practical implementation of the investigation. Such streamlined management of large data sets can be supported by implementing machine learning algorithms. We anticipate a more pronounced utilization of deep learning initially in massive data analysis and also in extracting concealed signals to complete high-resolution 3D imaging [83]. Additionally, adapting single-pulse XFEL time-resolved imaging to ultrafast electron sources may provide even higher spatial and temporal resolution, further enhancing our comprehension of these intricate processes. The development of new large-scale facilities is actively underway through international collaboration. This progress will significantly enhance accessibility for researchers from diverse fields, extending beyond X-ray or electron scattering, and provide a versatile experimental platform to explore strong light–matter interactions.

### Structural dynamics in liquids

For laser-based materials processing in liquids, including techniques of LAL, LFL, and LML, a key challenge lies in managing the intricate interplay between laser energy deposition, plasma generation, and the dynamic behavior of the surrounding liquid environment. The liquid medium not only mediates energy transfer but also plays a crucial role in influencing NP generation mechanisms, shock wave propagation, and bubble dynamics. Understanding the liquid dynamics is therefore essential for optimizing process parameters, improving reproducibility, and tailoring material properties for specific applications. Ultrafast optical techniques such as transient absorption spectroscopy (TAS) [84–87] have been applied to investigate the influence of the liquid environment on the energy relaxation processes of laser-excited NPs. While the TAS technique provides insight into the electronic properties of the NPs, it is, however, unable to reveal the response of the surrounding liquid to the excitation. To this end, the time-resolved scattering techniques based on X-rays and electrons are more appropriate because of their capability of visualizing atomic-scale structure evolution.

In this section, we focus on the time-resolved electron scattering technique, also known as ultrafast electron diffraction (UED). We review the fundamental principles of this technique and present an example of its application in studying structural dynamics in ionized liquid water.

Electrons and X-rays can both be used to structurally characterize the sample material and are often combined to best understand a sample. Electrons are sensitive to the electrostatic potential of the object and hence can probe both electrons and nuclei, whereas X-rays predominantly interact with the electron ingredient of the object. Furthermore, for samples such as liquid water, electron scattering probes both O–O and O–H intermolecular bonds, while X-ray scattering is specifically sensitive to the O–O bond distance [88]. However, time-resolved liquid scattering studies with the use of electrons have been hindered by their shallow penetration depth (*<*1 μm) compared to that of hard X-rays (*>*100 μm). Recently, the development of free-flowing liquid sheet jets has overcome this limitation by providing flat liquid sheets with thicknesses down to tens of nanometers [89–90]. The integration of this capability with a megaelectronvolt UED (MeV-UED) system has enabled electron scattering experiments for a variety of liquid-phase systems [88,91–94].

**Figure 4:**
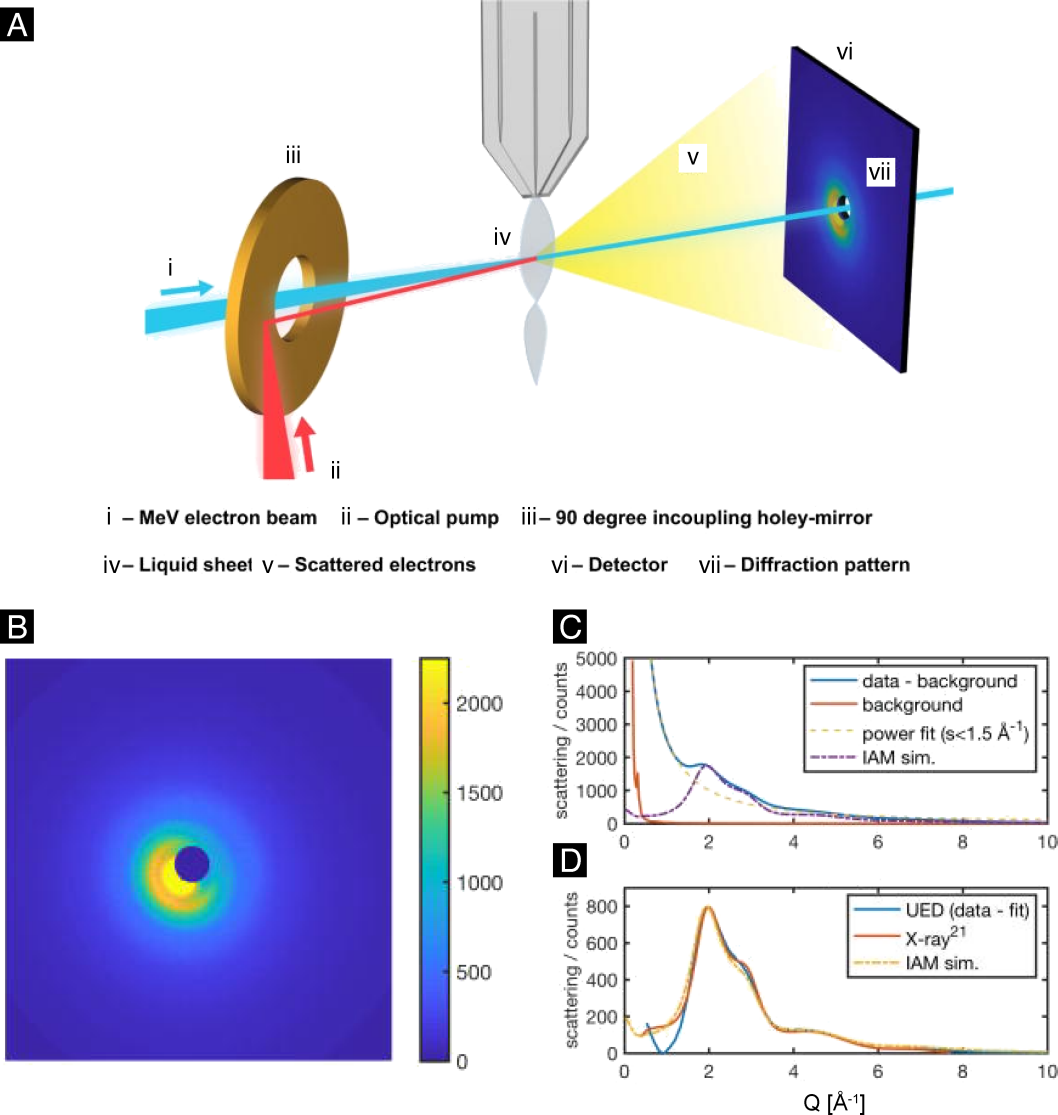
MeV-UED studies of structure dynamics in solution. (A) Schematic diagram of the experimental setup. An ultrathin liquid sheet was pumped by the fs optical pulse, and probed by a synchronized MeV electron beam. The scattered electrons were recorded as a scattering pattern at the detector. (B) An example of the electron scattering pattern for liquid water. (C) Azimuthally averaged scattering signal for liquid water at 290 K. (D) UED, X-ray and simulated elastic scattering curves of liquid water. Figure 4A–D was adapted from [91] (© 2020 J. P. F. Nunes et al., published by AIP Publishing, distributed under the terms of the Creative Commons Attribution 4.0 International License, https://creativecommons.org/licenses/by/4.0).

Figure 4A presents the schematic diagram of the liquid electron scattering experiments conducted at the MeV-UED facility of SLAC National Accelerator Laboratory. This setup features a high brightness, femtosecond electron beam operating at 3–4 MeV energies, coupled with a rapid-flow liquid sheet jet system, which ensures that the sample is refreshed before each pump–probe shot sequence. The scattering electrons of the liquid sample pumped by a synchronized femtosecond laser pulse are then recorded by a detector as a scattering pattern, which provides a momentum transfer range (denoted by *q*) reaching as large as 10 Å^−1^, cf. Figure 4B for the liquid water scattering pattern. Here *q* = 4πsinθ/λ, where θ is the scattering angle, and λ is the de Broglie wavelength of the electrons. Figure 4C shows the raw scattering intensity lineout of liquid water that is obtained by azimuthal averaging the scattering pattern. The raw scattering intensity lineout includes contributions from elastic scattering, inelastic scattering, and system-specific background. With the last two components removed, the total elastic scattering signal (Figure 4D) exhibits a distribution typical of amorphous materials with two major humps situated between 1 and 4 Å^−1^, consistent with the X-ray scattering measurement as shown below in Figure 9.

The total elastic scattering, *I*(*q*), can be expressed as the sum of atomic, *I*_at_(*q*), and molecular, *I*_mol_(*q*), scattering terms: *I*(*q*) = *I*_at_(*q*) + *I*_mol_(*q*). The contribution of the atomic scattering to the overall scattering intensity is given by the sum of all the elastic scattering amplitudes for all atoms in the system:


[1] $$I_{\text{at}}\left(q\right)=\sum_{i=1}^{N}{\left|f_{i}\left(q\right)\right|}^{2},$$

where *N* is the number of atoms in the system and *f_i_*(*q*) is the elastic scattering amplitude, also known as atomic scattering factor, for the *i*-th atom. As indicated by the above equation, the atomic scattering contribution does not contain any structural information, and its intensity depends only on the atoms present in the molecule. It is worth noting that the atomic scattering factor for electrons can be calculated via a Fourier transform of the atomic potential, while for X-rays it can be obtained using a Fourier transform of electron density. The two results are however interchangeable using the Mott–Bethe formula [95] given by:


[2] $$f_{i}^{\text{Electron}}\left(q\right)=\frac{1}{8\pi^{2}a_{0}}\frac{Z_{i}-f_{i}^{\text{X-ray}}\left(q\right)}{q^{2}},$$

where *a*_0_ is the Bohr radius, and *Z_i_* is the atomic number of the *i*-th atom.

The molecular scattering contribution is given by the sum of interference terms for all atom pairs in the system:


[3] $$I_{\text{mol}}\left(q\right)=\sum_{i=1}^{N}\sum_{j\neq 1}^{N}\left|f_{i}\left(q\right)\right|\left|f_{j}\left(q\right)\right|\cos\left(\eta_{i}-\eta_{j}\right)\frac{\sin\left(qr_{ij}\right)}{qr_{ij}},$$

where *f_i_*(*q*) and *f_j_*(*q*) are the elastic scattering amplitudes of the *i*-th and *j*-th atom, respectively, and η*_i_* and η*_j_* are their corresponding phases; *r_ij_* is the internuclear separation between the *i*-th and *j*-th atoms. The interference term of the molecular scattering gives rise to the modulation feature of the total elastic scattering intensity, while the atomic scattering forms a global baseline that decays exponentially with *q*. This is still true for unoriented molecules in liquids, which do not need to show long-range positional correlations, but only intramolecular fixed bond distances and intermolecular short-range correlation, which can be captured by the statistical pair distribution function.

In pump–probe experiments, the optical excitation results in molecular structural changes, thus the differential scattering signal with respect to the unpumped signal solely contains the molecular structural information: Δ*I*(*q*, *t*) = *I*(*q*, *t*) − *I* (*q*, *t <* 0) = Δ*I*_mol_(*q*, *t*). The molecular structural change can be retrieved through a differential pair distribution function (ΔPDF) given by:


[4] $$\Delta \text{PDF}\left(r,\;t\right)=\int_{q_{\min}}^{q_{\max}}\Delta sM\left(q,\;t\right)\exp\left(-kq^{2}\right)\sin\left(qr\right)\text{d}q,$$

where 
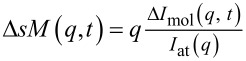
, and exp(−*kq*^2^) with *k* a constant is a damping function to suppress high-frequency artifacts generated by the truncation of Δ*sM*(*q*, *t*) at *q*_min_ and *q*_max_. What ΔPDF essentially reflects is the local structural change between the excited state and the ground state at different interatomic distances of the molecule. Positive peaks in ΔPDF indicate an increased probability of finding atom pairs at specific distances, while negative peaks reflect a decreased probability.

**Figure 5:**
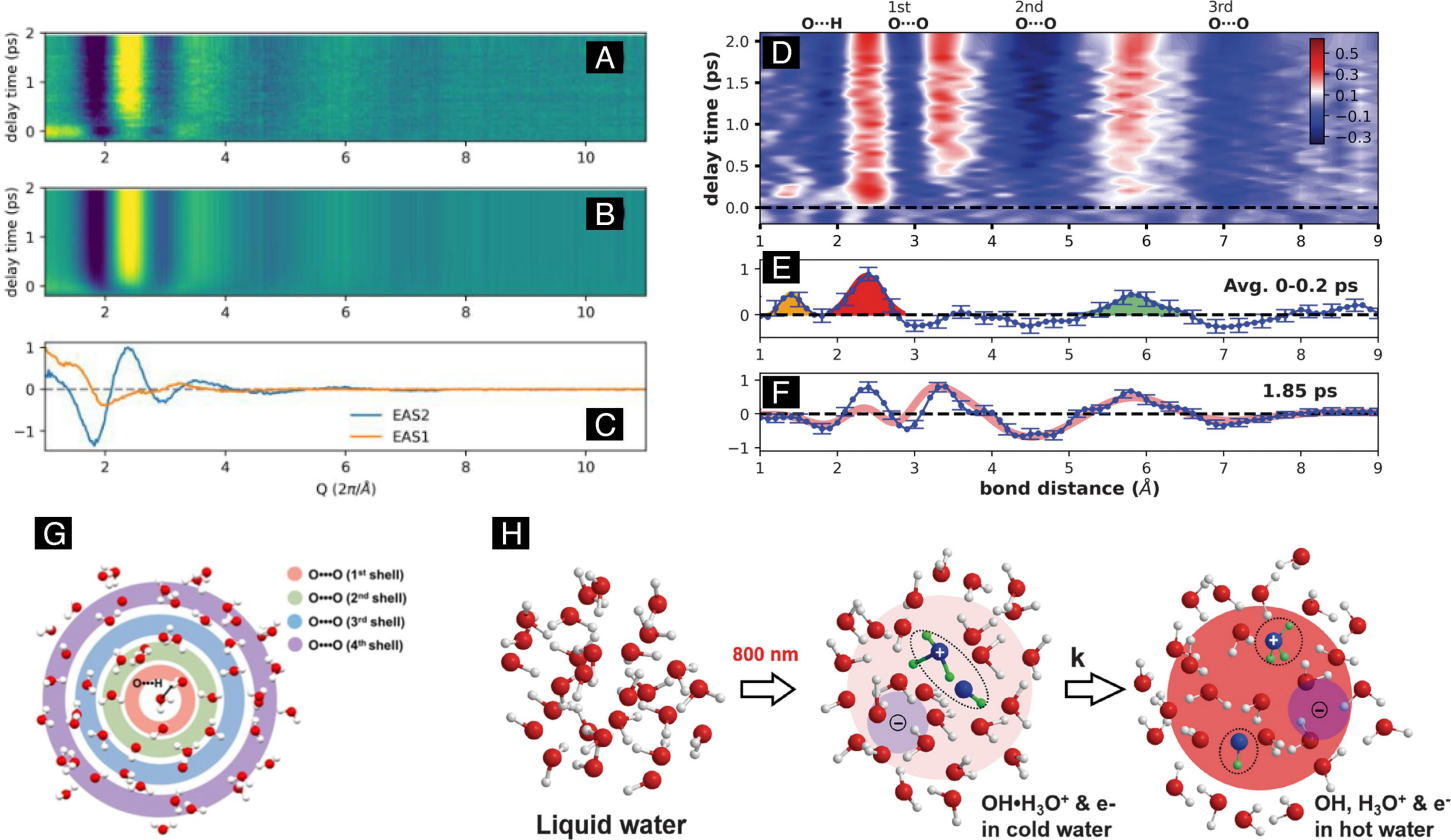
MeV-UED studies of ionized liquid water. (A) Experimental results of Δ*I*_mol_(*q*,*t*). (B) Reconstructed Δ*I*_mol_(*q*,*t*) from 2D global fit. (C) Evolution-associated spectra (EAS) from the global fit under the assumption of first-order kinetics (EAS1→EAS2). (D) ΔPDF(*r*,*t*) results of the ionized liquid water. (E, F) Selected experimental transient diffraction signals at averaged delay from 0 to 0.2 ps (E) and at 1.85 ps (F). The pink trace in (F) represents the ΔPDF between hot water at 610 K and water at 290 K from MD simulations. (G) Graphic representation of atom pairs in liquid water. (H) Cartoons showing the ionization of liquid water and the formation of solvated hydronium, hydroxyl radical, and electron, followed by the local thermalization through nonradiative relaxation. Figure 5A–F and H is from [94]. Reprinted with permission from AAAS. This content is not subject to CC BY 4.0. Figure 5G was reprinted with permission of The Royal Society of Chemistry, from [88] (“Structure retrieval in liquid-phase electron scattering” by J. Yang et al., *Phys. Chem. Chem. Phys.*, vol. 23, issue 2, © 2021); permission conveyed through Copyright Clearance Center, Inc. This content is not subject to CC BY 4.0.

Here, we showcase the work done by Lin et al. [94] investigating the early-time structural dynamics in the radiolysis of liquid water using the technique of time-resolved electron scattering (Figure 5). As context, the elementary reaction pathways in ionized water have been extensively studied [96]. Upon ionization, liquid water produces hydrated electrons and water cations (H_2_O^+^), which undergo proton transfer with neighboring water molecules at timescales shorter than 100 fs to generate hydroxyl radicals (OH) and hydronium cations (H_3_O^+^) [97]. Simultaneously, the ionized electrons in the excited p-like state undergo rapid solvation by neighboring water molecules in ≈50 fs or transition nonadiabatically to the s-like state at a similar timescale [98–100]. The hydrated and thermalized electrons then recombine with ion cores and hydroxyl radicals through geminate and non-geminate recombination processes, evolving on timescales ranging from tens of picoseconds to nanoseconds [100].

In this work, Lin et al. investigated the transient intermediate complex formed along the reaction pathways of ionized water and the corresponding structural dynamics following the proton transfer reaction. Time-resolved electron scattering proved to be an ideal technique for this study, since it provided sufficiently large momentum transfer and femtosecond temporal resolution required to capture the change in the bond distance of an ultrafast chemical reaction. These results would certainly contribute to the understanding of the liquid dynamics in laser excitation of solvated NPs, especially regarding the ionization and heating of the liquid water environment either directly by the laser electric field under high-intensity regime or through electron ejections from the NPs.

Figure 5A–C shows the result of Δ*I*_mol_(*q*, *t*) for the first 2 ps after laser excitation, with the corresponding ΔPDF result presented in Figure 5D–F. After laser excitation, ΔPDF clearly shows pair density reductions at radial distances corresponding to O···H hydrogen bond and the first three O···O coordination shells of neutral water. For better illustration, these atom pairs are highlighted in a virtual liquid water system as shown in Figure 5G. Another key feature with ΔPDF result is the appearance of new atomic-pair distances in between O···H and O···O bonds.

Equally interesting is the rapid change of these signals in the time domain, the underlying physical picture of which is provided in Figure 5H. Nonetheless, the transient structure averaged over the first 200 fs showed that two correlation peaks were formed at 1.4 and 2.4 Å, respectively, as shown in Figure 5E. These two peaks were attributed to the O···H and O···O bond distances of the solvated OH(H_3_O^+^) pair before separation. The experimental result showed that these features were short-lived and disappeared within ≈250 fs. This separation occurred because of the subsequent proton propagation from H_3_O^+^ to the outer-shell neutral water. The radical–cation pair dissociation process coincided with the heating of the water and contributed to the growth of signals at 3.4 and 5.8 Å at a later time. The overall change of the signal was observed to be steady after 500 fs. Interestingly, the experimental ΔPDF at a steady state could be well described by the temperature jump effect (Δ*T* ≈ 320 K) of neutral water, as shown by the agreement with the MD simulation result in Figure 5F. This implied that the rearrangement of the water molecular structure was dominated by the heating effect when the steady state was reached. These observations and the approach of analyzing the water structure provide an alternative pathway to understanding the energy dissipation pertinent to NP excitation in a liquid medium, as described in the next section.

The abovementioned results highlight the capability of UED in probing laser-induced structural dynamics in liquid-phase systems. In particular, its femtosecond time resolution and ability to access large momentum transfer enable real-time imaging of chemical bond dynamics. We anticipate that UED will complement X-ray scattering techniques in studying liquid dynamics, providing critical insights into energy dissipation processes in laser-excited NPs within a liquid environment. Potential applications of UED include investigating the non-thermal heating of water molecules surrounding NPs, which occurs on femtosecond to picosecond timescales, as well as examining the response of the water environment to heat transfer and the mechanical work performed by the expanding NPs.

### Nanoparticle excitation in an ensemble and energy exchange with the medium

With the latest advances in ultrafast imaging down to an atomic scale (see the section on single-particle imaging), it became possible to visualize photoreactions in individual particles [68,101–102], including internal strain distribution or polar vortices [103]. In addition to focusing on single, selected particles it is also possible to perform such experiments as massively repeated single-shot imaging to cover heterogeneous ensembles [104]. Still, most of the laser processing in application-relevant cases deals with continuous cycling of a large ensemble of particles, which in contact with a liquid medium involves cooperative phenomena, such as heat dissipation, evaporation, creation of shock waves, or reorganization of formed products like fragmented clusters to new objects by ripening. Therefore, it is important to understand and quantify such processes that may occur concomitantly and will affect each other.

**Figure 6:**
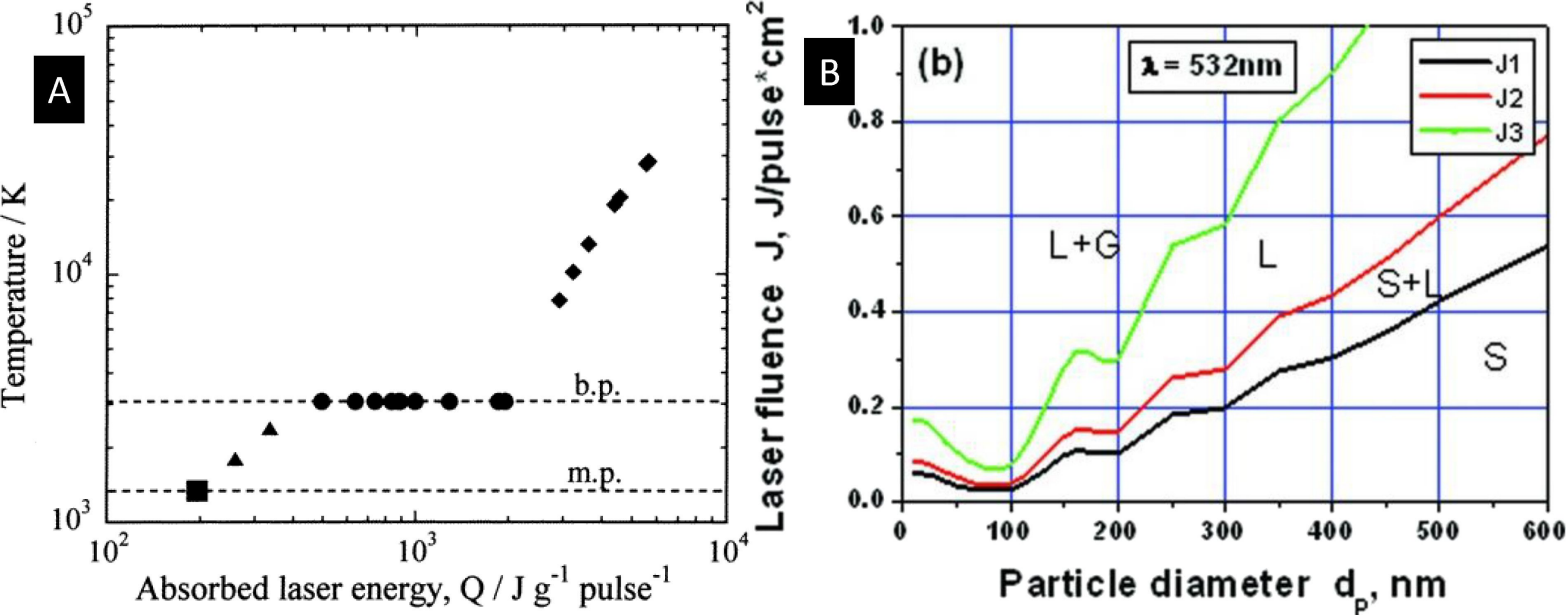
(A) Estimation of temperature change of slowly heated gold nanoparticles as function of absorbed laser energy. (B) Calculation of the laser fluence required to convert excited gold NPs into the (partially S+L) molten state (L) or partially evaporated state (L+G) with 532 nm radiation with 10 ns pulse length as function of particle diameter. Figure 6A was reprinted with permission from [46], Copyright 1999 American Chemical Society. This content is not subject to CC BY 4.0. Figure 6B was reproduced from [47], A. Pyatenko et al., “Mechanism of pulse laser interaction with colloidal nanoparticles”, *Laser & Photonics Reviews*, with permission from John Wiley and Sons. © 2013 by WILEY-VCH Verlag GmbH & Co. KGaA, Weinheim. This content is not subject to CC BY 4.0.

First, we develop a basic hypothesis of how laser irradiation with significant energy deposition into the system of interest (for example an aqueous colloid or solid surface in contact to liquid) should lead to restructuring. In a simple model, the laser energy is converted into heat that will be localized in the absorbing part within the laser penetration depth. Absorption is linear as expressed by the (known) absorption cross section. In the model by Takami et al. [46], which is expected to hold from nanosecond pulse excitation, the absorbed energy by the laser pulse is converted into lattice temperature by taking the material heat capacity into account. Figure 6A shows the temperature of heated gold NPs as a function of the absorbed laser energy. Once the temperature reaches the melting point, melting sets in. The amount of molten material is given by the additional available energy beyond the heat capacity in the solid state to account for latent heat of melting [47,105], see calculations shown in Figure 6B. After full melting, the temperature of the particle will increase further until the evaporation threshold is reached. Evaporation again requires additional energy to overcome heat of fusion. Only evaporation can lead to expulsion of material and formation of clusters in this context via condensation of the vapor. Further processes that will be described in the laser ablation section, such as phase explosion, do require fast heating for the material to leave the binodal of liquid–gas coexistence and reach the critical point or the spinodal line [38].

This model offers a conceptually straightforward access to classifying structure formation according to the maximally reached temperature after photoexcitation. However, it should not be taken literally, if the excitation is done by shorter pulses in the picosecond range, where a substantial difference between electron and lattice temperature can take place. At the same time, the melting process on the nanoscale will not be described by a steady-state process as on macroscopic length scales. Melting as a heterogeneously driven phase transition may require nucleation, which sets the timescale for the transition to the liquid state to some picoseconds in defective systems to some hundreds of picoseconds in single-crystalline samples, which lack nucleation sites. Thus, a transient overheating above the melting point could even take place, which lasts for 100 to 200 ps. On longer timescales, heat dissipation can set in during the heating with nanosecond or longer pulses. Heat dissipation in small NPs is strongly size-dependent and can be as short as 100 ps for small particles below 20 nm. Steady-state evaporation is also inefficient for mass transport on a picosecond-to-nanosecond timescale that is of interest here. Thus, thresholds for ablation and fragmentation will shift to energies far above the energy required to reach the boiling point of the irradiated material.

With these complications in mind, it is useful to take a closer look at the energy distribution pathway. In almost all cases of linear absorbing materials, the laser light interacts first with electrons that are excited into a state far above the Fermi level. These electrons thermalize within hundreds of femtoseconds to a temperature much higher than the lattice temperature [106]. Under such a highly nonequilibrium condition, hot electrons transfer their energy to the cool lattice of NPs through the electron–phonon coupling process. As the electron heat capacity is temperature-dependent in simple metals [28] (and the scattering cross section decreases with temperature), the cooling of the electron gas tends to slow down with increasing excitation density [107]. A well-known case is gold with exceptionally weak electron–phonon coupling, such that the complete cooling of the electron gas could last for tens of picoseconds [23,108]. Therefore, excitation with short pulses of picosecond duration or shorter would display transient differences between electronic and phonon subsystem. This selectively heated electron gas can instigate processes other than purely thermal effects. Among these are electron emission [109], near-field forces of the plasmon resonance on the surface, pressure effects due to an expanding electron gas [25,27], or spatial spreading of fast electrons [110–111]. In general, with femtosecond excitation a large fraction of electrons (of the order of 10%) can be excited, which would lead to changes of the interatomic forces faster than electron–phonon coupling. This has been seen in semiconductors [24,112] in particular.

The electron–phonon coupling will increase the temperature of the lattice, which will undergo phase transitions when sufficient thermal energy is coupled to the lattice. Depending on the energy density condition the lattice reaches, phase transitions can result in coalescence of multiple NPs [15,105] or in transformation of NPs into vapor, atomic clusters, and small molten NPs. In both cases, the temperature difference between the hot NPs and the surrounding liquid drives heat transfer from the NPs to the liquid environment. This heat transfer process impacts the thermal equilibrium within the NPs, thereby shaping their phase evolution pathway. Furthermore, the non-thermal effects that were described above can also play a role in the energy dissipation of the photoexcited NPs.

Thus, a description and verification of putative processes that lead to structure formation, such as NP formation after laser ablation or particle breakup in laser fragmentation requires sensitive tools for detecting and discerning the largely co-evolving processes. Given a timescale for electron temperature equilibration of few picoseconds and one for diffusive ripening processes of minutes to hours, the time-resolved tools require a temporal resolution that spans many decades. Early experimental efforts focused on probing energy dissipation from metallic NPs to their liquid surroundings, employing TAS [84–87]. This technique operates by exploiting the photoexcitation of free electrons within the NPs, which modulates the absorbance spectrum of the probe light. As the transmission through a macroscopic suspension containing NPs or reflection from a photoexcited NP is determined by absorption as well as scattering, several structural processes may contribute to a change in signal.

Metal NPs show SPR linked to coherent excitation of electrons in the conduction band. Its position and width are given by the dielectric functions of metal and medium and are described by Mie theory [113]. Mie theory allows one to derive absolute absorption and scattering cross sections of primarily spherical or core–shell objects, but also different shapes by extensions of the Mie–Gans [114] theory or numerical approaches [115]. The subsequent pressure and temperature conditions emanate from the close interaction of the excited particles with the medium. The width of the SPR reflects the coherence time of this oscillation. This dephasing time amounts to a few femtoseconds, leading to a plasmon resonance width of several tens of nanometers [116]. In turn, a strongly excited electron gas leads to an increased scattering rate, which can be detected as plasmon broadening in TAS experiments.

A change of electron temperature manifests itself as a “bleach” in the surface plasmon band, accompanied by a positive transient absorption signal near the bleached region, signifying the broadening. The decay dynamics of these signals are dictated by electron–phonon coupling within the particle and phonon–solvent interaction at the particle surface, hence informing about the overall energy relaxation timescales.

**Figure 7:**
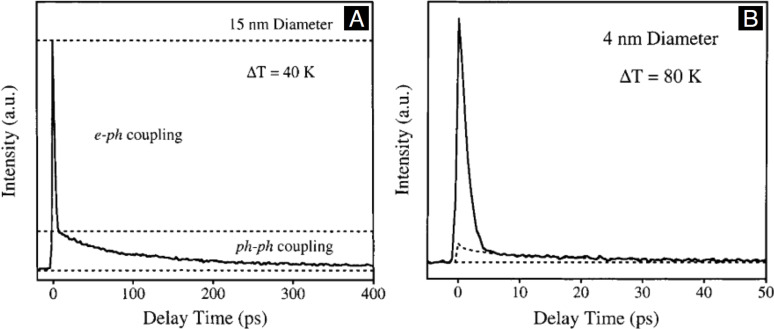
Ultrafast transient absorption data measuring the heat transfer from Au NPs to the liquid environment. (A) Measurement result of time-resolved absorbance intensity for 15 nm diameter Au NPs. (B) Result for 4 nm diameter Au NPs. For both cases, temperature rises of the laser-heated NPs are indicated inside the plots. Figure 7 was reprinted with permission from [87], Copyright 2002 American Chemical Society. This content is not subject to CC BY 4.0.

The application of the transient absorption technique was nicely demonstrated in a work by Hu et al. [87], in which they measured solvated NPs of varying sizes and determined the effect of size on the rate of energy relaxation. As shown in Figure 7A, the transient bleach results for larger NPs showed two distinct decay regions: (i) an initial fast decay due to electron–phonon coupling within the particle and (ii) a slow decay due to heat dissipation from the particle to the environment. It was found that the relaxation times of heat dissipation were proportional to the square of the radius, but did not depend on the initial particle temperature. For smaller particles, as shown in Figure 7B, the results revealed that the timescale for heat dissipation was comparable to that of electron–phonon coupling. This suggests that significant energy loss had occurred before electrons and phonons reached thermal equilibrium within the particle. It was proposed that the solvent molecules directly interact with non-thermal electrons at the particle surface, particularly in very small particles.

In fact, the relatively well-defined geometry of NP colloids that leads to excitation localization and uniformity as well as the understanding of the optical response of the SPR helped to clarify specifics of electron dynamics and coupling to the phonons and close boundaries. Valleé and Del Fatti et al. [117–118] and others [119–120] addressed size effects of electron dynamics as well structural relaxations by these model systems.

Electron emission has been verified in many nanoscale systems in vacuum, where the emission is accessible. It is less straightforward to directly detect non-thermal effects as electron emission in liquid media. Mafuné and coworkers managed to record the spectral signature of solvated electrons in water after massive excitation of silver NPs [121]. Also, light emission can be harnessed to access the local temperatures of molecules [122–123] or by using reporter fluorophores that are sensitive on temperature [124].

**Figure 8:**
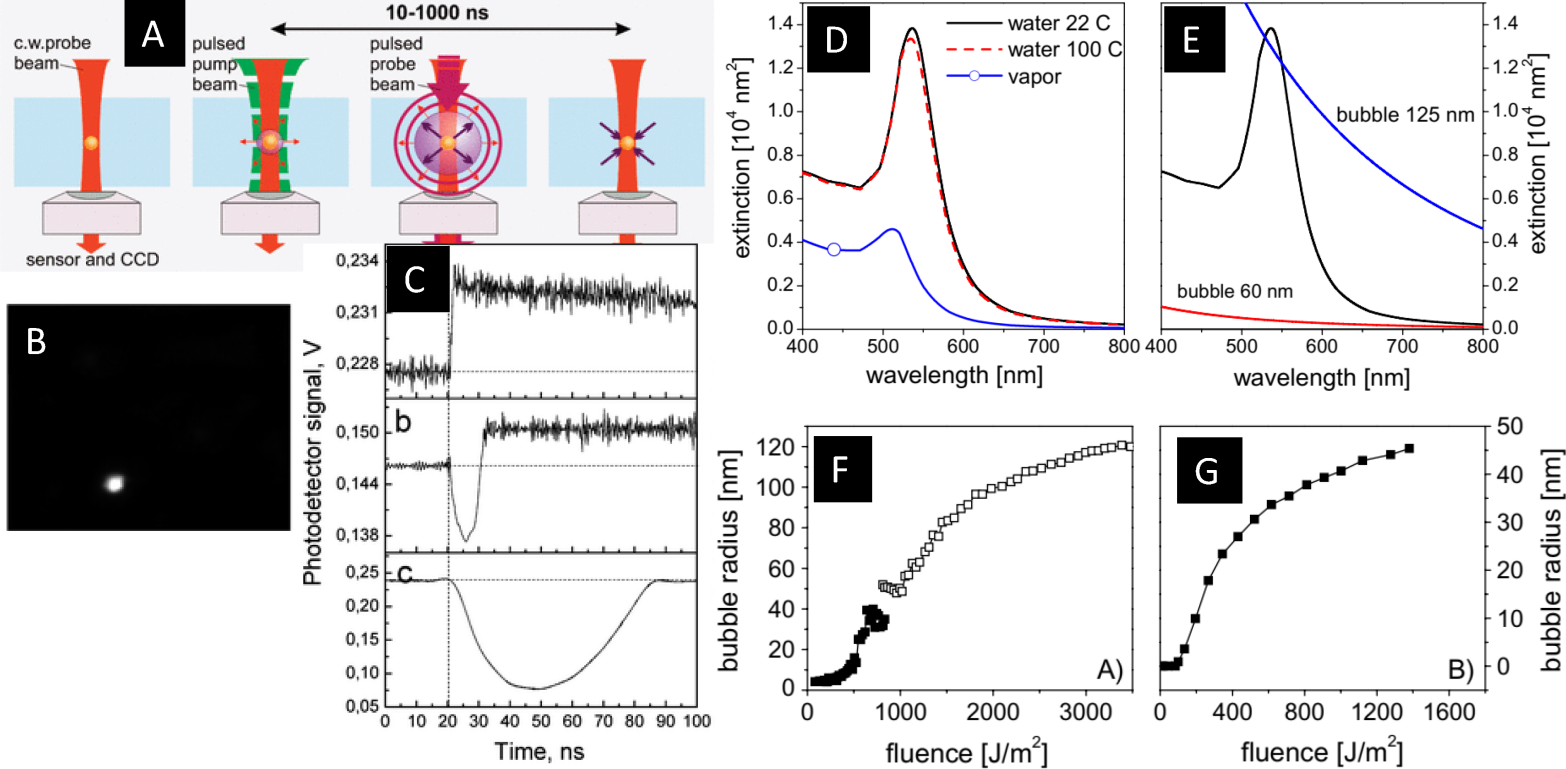
Examples for time-resolved optical studies of gold NP excitation. In (A) TAS with a pulsed pump beam and a continuous-wave probe beam is demonstrated for absorption contrast and imaging contrast, with a selected scattering image for a generated nanobubble (B) and transient transmission traces (C) for increasing laser fluence. Changes in optical extinction of 60 nm gold particles in water at 22 °C, 100 °C and in vapor (D) as well as additional contribution by light scattering from 60 and 125 nm vapor bubbles (E). Bubble sizes on excited NPs as a function of the applied fluence for nanosecond pulses (F) and femtosecond pulses (G). Figure 8A–C was adapted with permission from [127], Copyright 2010 by American Chemical Society. This content is not subject to CC BY 4.0. Figure 8D–G was reprinted from [125] (“Thermodynamics of nanosecond nanobubble formation at laser-excited metal nanoparticles”, © 2011 A. Siems et al., published by IOP Publishing, distributed under the terms of the Creative Commons Attribution-NonCommercial-ShareAlike 3.0 Unported License, https://creativecommons.org/licenses/by-nc-sa/3.0/). This content is not subject to CC BY 4.0.

Structure formation, such as lattice heating, melting, or shape transformations, is normally considered to set in with electron thermalization with the lattice. Nevertheless, the hot electron gas can exert structural forces on the lattice even before thermalization. Gold or silver NPs in particular have displayed non-thermal effects upon excitation, such as directed fragmentation or near-field ablation [26,52]. While electronic excitation is easily detected by TAS, the various structural responses of NPs are weaker and ambiguous in TAS. While elastic oscillations of NPs are identified in periodic modulations of the plasmon resonance due to the change of electron density, other responses, such as particle heating or melting, are less characteristic. In fact, pure heating of gold particles leads to a very small change in SPR position [120], which is even modulated by an eventual change of the temperature of the surrounding liquid through a change of the refractive index [125]. Light scattering becomes relevant if the structures exceed a size of a fraction of the wavelength [126–128]. Figure 8A shows several schemes of exciting NPs by focused laser beams for excitation and probing. Both coaxially and side illumination allow to image the scattering emitted from induced nanobubbles [127] shown in Figure 8B.

The SPR of liquid metal particles, such as gold, should drastically change because of the change of the dielectric function. Nevertheless, the melting transition has never been detected spectroscopically on an ultrafast timescale. In thin-film geometry, the optical reflectivity has been reliably assigned to film melting [129–130]. Optical reflectivity is discussed in more detail in the following chapter.

Additionally, if the NP temperature is high enough, the heat released to the liquid within a few tens of picoseconds can be sufficient to form vapor bubbles around the particles. In this case, the refractive index changes strongly, which leads to a strong shift and dampening of the SPR. A calculation of the extinction for gold NPs in water for various water temperatures, including water vapor is depicted in Figure 8D, as well as the extinction of vapor bubbles (Figure 8E). Although the change of water temperature imposes only a minor change on the SPR, the formation of a bubble drastically reduces the SPR height in a fashion similar to NP melting. For bubbles exceeding a diameter of 60 to 100 nm, the transmission loss due to scattering becomes relevant, which in sum can lead to both positive and negative transmission changes, as shown in Figure 8C. Therefore, TAS at this point is no longer an unambiguous probe for the resolution of such effects [131]. Nevertheless, the bubble formation as a threshold process can still be identified through the characteristic change in transient absorption, first through plasmon dampening and with increasing bubble size through the increase in scattering once the bubble reaches some 100 nm in diameter. A quantitative analysis of the change of the TAS signal as compared to Mie calculations can in some cases even reproduce the bubble formation threshold as well as bubble sizes [125,128]. Figure 8F shows that bubble size growth with laser fluence can be extracted with good correlation to X-ray results (Figure 8G).

Structure formation, such as LAL or LFL in NP colloids and on surfaces, involves all steps of particle heating, melting, evaporation or bubble formation of the surrounding liquid; TAS alone is not sufficient to identify all these steps. Scattering methods with short wavelength allow for multiple length scales to be resolved down to the atomic scale. UED, for instance, can resolve atomic-scale structure formation [65,132] with picosecond time resolution, as described in the section “Structural dynamics in liquids”. The high interaction cross section is suitable for minuscule material quantities, such as nanostructures [23], surface phenomena [133], or gases [134], but poses challenges regarding the bulk condensed phase. X-ray scattering, in contrast, penetrates matter deeper due to a cross section that is 10^5^ times smaller. Brilliant sources for pulsed X-rays are modern synchrotrons with pulse lengths of about 60–100 ps at high repetition rate of some megahertz, which can be used in time-resolved experiments by synchronized lasers to selected subpulses in the accelerators that produce X-rays [135]. X-rays probe the electron density distribution that can be expressed as pairwise summation of pairs of scatterers of scattering length *f_i_*(*q*), see Equation 3, leading to the general notion of the scattering distribution in *q*-space being the Fourier transform squared of the real-space scattering length distribution. As this scattering length is well known, scattering can be described quantitatively in units of the electron scattering strength *r*_0_ = 2.8 × 10^−15^ m.

**Figure 9:**
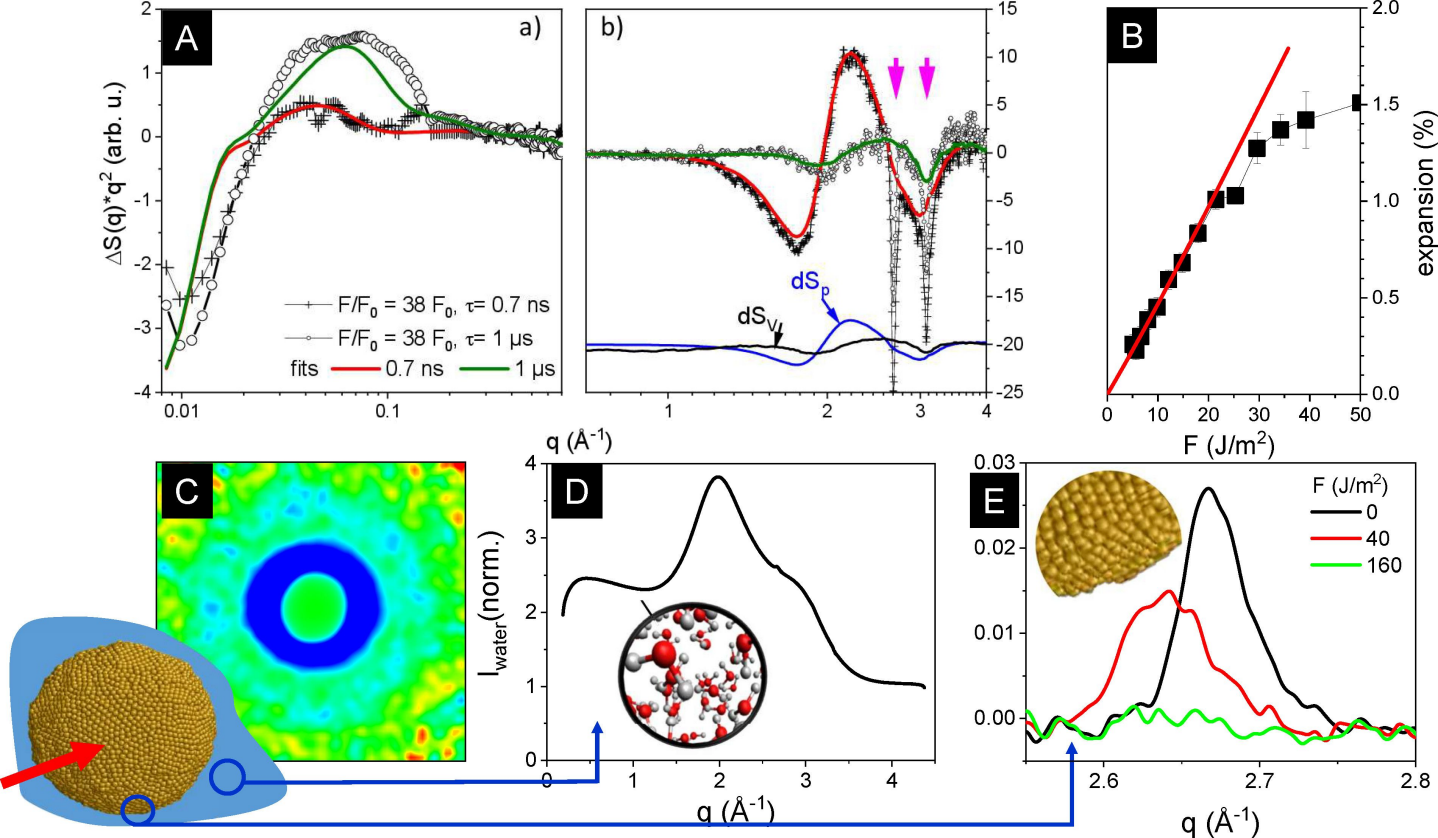
(A) Change of X-ray scattering distribution of a laser-excited gold particle suspension in water at 400 nm over a wide scattering vector range *q*. The lowest range of *q* (0.01–0.1 Å^−1^) relates to the largest structures and their changes, such as fragmentation of the initial 50 nm particles or formation of concentric vapor bubbles, with the corresponding 2D difference scattering distribution shown in (C). Fragmented small particles appear in a large *q* range of 0.1–0.5 Å^−1^. The wide-angle scattering region from 0.5 to above 4 Å^−1^ is dominated by water scattering (D), which changes distinctly upon change of thermodynamic parameters. The crystalline lattice of gold produces narrow powder peaks corresponding to the (111) and (200) lattice planes, which react upon heating and/or melting of the lattice. The arrows in (A, part b) indicate the loss of powder scattering due to particle fragmentation. A shift in peak position is indicative of particle heating and serves as a thermometer with a linear shift with applied fluence in (E). Figure 9A was reprinted with permission from [137], Copyright 2025 American Chemical Society. This content is not subject to CC BY 4.0. Figure 9C was adapted with permission from [136], Copyright 2024 American Chemical Society. This content is not subject to CC BY 4.0. Figure 9B, D and E was newly plotted using data from [136].

NP materials such as suspensions represent a heterogeneous system of several structural length scales, all of which will be detected in an appropriate geometry and mass relation. Figure 9 shows an example of different structural features that can be observed as function of *q*-scale and thus the inverse length scale after photoexcitation of a aqueous suspension of 53 nm gold particles. The particles only occupy a mass fraction of 0.02%. Figure 9A displays the relative changes in scattering when the suspension is irradiated by 1 ps laser pulses at the interband transition at 400 nm. This difference, Δ*I*, includes negative and positive contributions depending on the formation, disappearance, or shift of structural features. The Fourier transform of the complete particle leads to SAXS at low scattering vectors corresponding to π/*D* with *D* being the particle size. In Figure 9C such a 2D depiction of ΔI*q^2^ is shown in false colors, where blue areas indicate negative difference and red areas indicate positive difference. At the lowest observable *q*, the change is negative, qualitatively indicating that the largest structure is lost during photoexcitation because of particle fragmentation [136]. Increased intensity at intermediate *q* (0.1–0.6 Å^−1^) reveals new structure formation on the 1 to 5 nm scale, which is caused by the formation of clusters from the fragmentation process.

While the gold content in the sample is very low, the scattering over a wide range in *q* (0.5 to above 4 Å^−1^) is dominated by liquid scattering of water (Figure 9D). Because of the ordering in the liquid, the preferential distances of oxygen in water cause the maxima around 2.0 and 2.8 Å^−1^ [138], as explained in the liquid structural dynamics section. As discussed above, water is not a pure spectator of the gold excitation but reacts by heating, bubble formation, compression, or expansion, all of which will modify the scattering function. In the limit of weak perturbation of the structural degrees of freedom, the change in the scattering distribution can be considered as a sum of stationary functions ∂*S* related to the independent thermodynamic variables temperature *T*, pressure *p*, and density ρ [139–140]:


[5] $$\Delta I\left(\rho ,T,q\right)={\frac{\partial S}{\partial T}|}_{\rho }\cdot \Delta T+{\frac{\partial S}{\partial \rho }|}_{T}\cdot \Delta \rho .$$

These stationary functions are characteristic for any liquid and can be derived in control experiments. Thus, the analysis of change in water scattering offers an access to the thermodynamic state of the medium that hosts the NPs. The functions 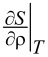 and 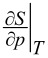 are displayed in Figure 9A and are used to model the water scattering changes. Most prominently, bubble formation will lead to a compression of the adjacent liquid water, such that the amplitude Δ*p* of the function 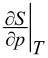 will be proportional to the bubble volume [141–142]. Finally, the crystalline lattice of the gold particle produces Bragg peaks that scale in angular position with the inverse of the lattice parameter *d*. These peaks are registered as powder rings, as the ensemble of NPs is not oriented in the liquid. The width of the peaks depends inversely on the crystal grain size, while the integrated intensity scales with the crystalline volume. In the data displayed in Figure 9, the experimental resolution is the limiting factor to the peak width.

The powder scattering opens direct access to the thermodynamic state of the NPs with the temperature increase leading to a change of the lattice parameter,


[6] $$\frac{\Delta d}{d}=\alpha \Delta T,$$

due to thermal expansion with expansion coefficient α (which could be temperature-dependent) and melting causing a loss in diffraction intensity. The change of the extracted (111) powder peak position with increasing fluence of excitation is shown in Figure 9E. The peak shift at 40 J/m^2^ can be converted into temperature change by using thermal expansion coefficient and latent heat of gold [30,136,143]. At 160 J/m^2^, the peak has completely vanished, proving melting of the particles due to the absorption of energy. A quasilinear variation of the excitation fluence immediately after photoexcitation (delay 60 ps, the experimental time resolution, see Figure 9B) shows that this assumption holds over a range of at least 1% in expansion. Note that gold at the melting point of 1360 K would display an expansion of 1.79%. This is remarkably similar to the behavior of the gold particles, where expansion is limited below 1.6%, and loss of powder scattering coincides with reaching this limit [33,136]. Thus, a threshold fluence *F*_0_ can be defined to reach the melting point of the particles by extrapolating the quasilinear expansion with fluence to the critical total expansion at the melting point. This procedure serves to find an internal calibrant that defines the energy uptake scale in a photoexcitation experiment. This can turn out to be important if either the absorption cross section is not known because of sample modifications or experimental boundary conditions such as fluence distributions or fluctuations are present. Transient bleaching or enhancement of absorption cross section would as well cause a discrepancy between measured static cross section and energy absorption in a pulsed laser field [14,144].

All the different hierarchies of structure change due to energy dissipation are accessible in a time-resolved X-ray experiment. The problem of describing the laser fragmentation process is manifold. First, the energy scale has to be quantified to understand whether the process is thermally activated or governed by non-thermal forces. Second, the evolution of length scales needs to be resolved to quantify the grade of damage to the initial particles and the formation of new species such as atoms, clusters, and particles. Finally, the amount of interaction with the environment needs to be highlighted, which includes heat transfer or redox reactions. Earlier simulations have given a real-space impression of how fragmentation could evolve in a photoexcited NP. MD simulations by Zhigilei and Garrison [36] demonstrated that isolated (van der Waals-bonded) NPs can be fragmented by stress confinement for short pulses relative to the acoustic relaxation time (15 ps for 100 nm particles), while excitation with relatively long pulses can lead to explosive thermal decomposition or phase explosion. This is also accompanied by an increase in the fluence required for fragmentation. Delfour and Itina [39] explored fragmentation by ultrashort laser pulses (0.15 ps) both by thermal decomposition and Coulomb instability (see Figure 1H). The latter can destabilize smaller particles by photothermal ejection of electrons, which induces shape instability [145] in the liquid state. For larger particles, a combination of stress-induced and thermal explosion is corroborated. Finally, Huang et al. [44–45] developed a detailed model by large-scale simulations of gold particles excited in water environment, including heat transfer and bubble formation. Excitation of 20 nm particles by 10 ps laser pulses clearly leads to a thermal fragmentation scenario, which evolves in different regimes from a partial (weak) fragmentation with formation of large and small fragments to a strong phase explosion that is described by a spinodal process. Moreover, the role of water was pointed out; water absorbs heat and also suspends atoms and small clusters after rapid cooling, but repels ejected larger, hot particles that eventually would reform a large particle at the location of the initial particle. This is ascribed to the so-called inverse Leidenfrost effect, whereby hot particles that reach the vapor–liquid boundary are repelled by the formation of additional vapor in a similar way to the way water droplets are repelled from a hot surface in the common Leidenfrost effect, or on heated NPs [146]. As a result, fragmentation seems to be less efficient when only regarding the final products rather than resolving intermediate structures.

Several experimental studies have addressed the fragmentation process both by recording transient dynamics and carefully analyzing NPs generated after the fragmentation [147]. Werner et al. [51] postulated a Coulomb instability [145,148] by transient increase of absorption due to formed nanoclusters and the post-mortem TEM analysis (see Figure 1B–E). Melting could also be identified in the final state by the rounding of initial facets on the particles and blue shift of the SPR [149–150]. Other studies focus on the polarization effect on fragmentation, which is indicative of directed fragmentation by forces induced by the incident laser polarization [151–152]. The notion is that field emission of electrons forms the force field for directed fragmentation and collection of debris [52]. Also, direct material ejection by the near-field has been described [26] as anisotropic fragmentation channel in the absence of particle melting.

**Figure 10:**
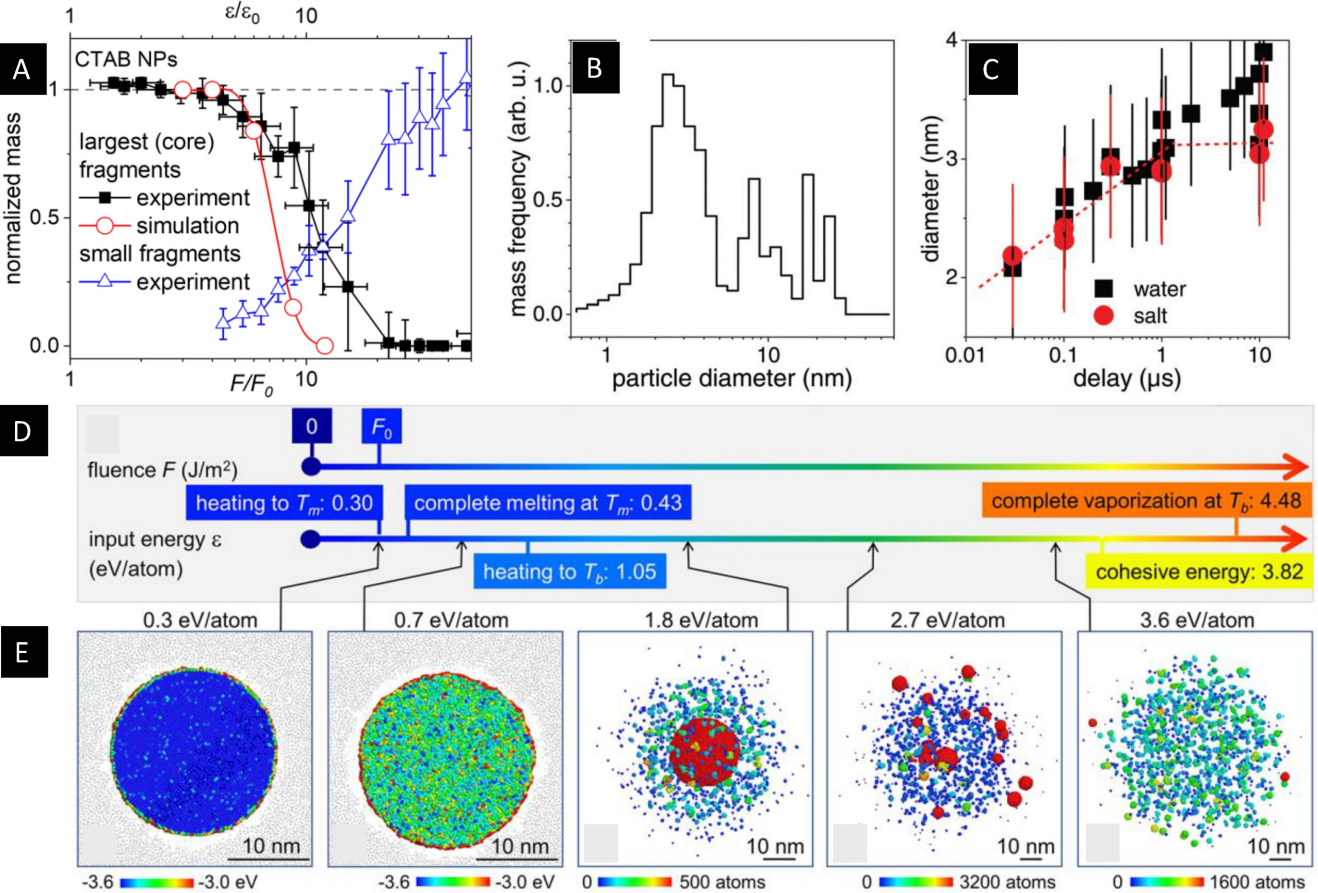
Caloric progress of gold NP heating and fragmentation as result of picosecond-resolved X-ray scattering experiments (A–C) and simulations (E) as function of laser fluence *F* and deposited energy per atom in the experiment and simulations, respectively. The scale of energy deposition is scaled by the fluence *F*_0_ or energy per atom ε_0_ to reach the melting point (energy scale in D). (A) The initial spherical particles of 44 nm diameter stay intact after undergoing a melting transition above an *F*/*F*_0_ of 1.43 up to 4 (higher than reaching the boiling point *T*_b_ at 1.05 eV or *F*/*F*_0_ = 3.5) at a 1 μs delay (400 nm), before being reduced in size gradually to vanish at *F*/*F*_0_ = 15. At the same time, clusters of 2 to 3 nm in diameter appear to end in complete fragmentation when the initial particles have vanished. (B) Fragment sizes are found to cluster around 3 nm with some remaining large fragments upon incomplete fragmentation at 532 nm. (C) The fragmented clusters would ripen through fusion and addition of active species on a microsecond scale, which can be partly suppressed by salt addition. (D) Mapping of the fluence scale as established in the experiment through identification of *F*_0_ on the energy scale of nanoparticle heating in the MD simulations including melting temperature, *T*_m_, and boiling temperature, *T*_b_. (E) Final state of simulations of 20 nm particles with increasing energy deposition, which reproduce the observations of complete recrystallization below 0.7 eV/atom followed by weak phase explosion (1.8 eV/atom) and formation of large fragments (2.7 eV/atom) that merge again (2.7 eV/atom) and finally full fragmentation through a strong phase explosion at 3.6 eV/atom. The color coding shows the temperature for 0.3 and 0.7 eV/atom and the cluster size in number of atoms for 1.8–3.6 eV/atom. Figure 10A, D and E was adapted with permission from [136], Copyright 2024 American Chemical Society. This content is not subject to CC BY 4.0. Figure 10B,C was adapted from [142] (“In situ structural kinetics of picosecond laser-induced heating and fragmentation of colloidal gold spheres”, © 2020 A. R. Ziefuss et al., published by RSC, distributed under the terms of the Creative Commons Attribution 3.0 Unported License, https://creativecommons.org/licenses/by/3.0/).

In recent multiscale time-resolved X-ray experiments [136,142] the question of structural fragmentation pathways and mechanism was tackled by the information on size evolution by SAXS measurements (Figure 10A,B) resolving the timescale of reactions as well as the energy, respectively heat distribution in the system. The latter is central for allowing to match the change of energy absorption by a variable laser fluence with the large-scale simulations [45] as indicated in Figure 10D. The matching of absorbed energy ε in the simulations and the applied fluence *F* in the experiment is done at the point where the melting transition is reached, which is termed *F*_0_ and easy to identify via thermal expansion as explained above.

Under the premise that thermal heating of the excited NPs is directly proportional to the applied external fluence, one can relate the structural reactions found at a given fluence scale to the structural snapshots from the MD simulations (Figure 10E). In particular, the number and sizes of fragments can be deduced from the experiment and compared to the simulation. Figure 10A reveals that the change of the size of the initial particle and the number and sizes of the produced fragments follow a characteristic change as a function of the fluence. The gold NPs survive laser excitation without damage, even if the fluence allows for transient melting. Only at 3–4*F*_0_ the particles start to get reduced in size. This is explained in the MD simulations by a weak surface evaporation and beginning of partial fragmentation. Although at higher fluence the MD simulations show a fragmentation in pieces after increasing the internal energy at 9ε_0_ these fragments fuse again after the mentioned inverse Leidenfrost effect. Thus, a full fragmentation with formation of uniformly sized clusters is achieved only at 12ε_0_ or 12–15*F*_0_ in the experiment.

The aspect of necessary timescales in the scattering experiments needs to be addressed. If the formation of a NP or a spatial distortion of a certain size develops with the speed of sound, this would set a shortest timescale worth resolving, which lies in the range of 3–30 ps for formed objects of 10–100 nm diameter. Thus, few picoseconds are appropriate to resolve the nascence of nanoparticles in an ensemble, with the 60 ps available at synchrotrons coming close [135]. However, single-particle experiments can take advantage of a much higher time resolution, if structural changes within single addressed particles happen on a sub-picosecond timescale as discussed above. At the same time, detection of small clusters in a diluted system such as in suspensions or liquids during LAL is limited because of the rapid decrease of the scattering cross section (scales with the volume *V*^2^). In time-resolved SAXS, this limit has been seen at about 1 nm in diameter for gold [136].

The experiments also show that the transient structuring is by far not the final state of the fragmentation process as the clusters are still at close distance (Figure 10E) and can aggregate again. In fact, ripening is found to occur on the microsecond timescale and will lead to a different final state, which could be observed in static analysis of the final state. Manipulating this ripening therefore requires intervention on a fast timescale or a strong dilution of the particles through fragmentation. The addition of electrolytes was shown to be efficient in collecting the smallest possible clusters after fragmentation [12]. Alternatively, supporting the clusters on a substrate [153] or embedding in a matrix [52] could also reduce ripening.

### Large-scale optical probing of laser ablation dynamics in liquids

In contrast to the fragmentation of NPs, typically homogeneous bulk samples are excited by a laser source during LAL to produce larger amounts of NPs. Here, the diameter of the laser-excited area lies typically in the range of a few tens of microns because the geometrical dimension of the optical setup and liquid cuvettes demand focusing distances of a few hundred millimeters. Since the dynamical processes initiated after laser pulse impact occur on timescales ranging from picoseconds to nanoseconds, with the fastest processes occurring on a sub-picosecond timescale, a high temporal dynamic range combined with high temporal resolution is of paramount importance for the experimental investigation of laser ablation. Optical probing methods fulfill this criterion with the disadvantage of a lower spatial resolution of several hundred nanometers compared to X-ray examination because of their longer wavelength. A key advantage of optical probing lies in its ability to observe the temporal evolution of the laser ablation process, either in normal incidence, which provides quantitative information on optical reflection and absorption, or perpendicular to the laser excitation axis by, e.g., shadowgraphy. Both enable the validation of existing models and support a deeper understanding of ablation dynamics. In the following, we will start with a brief introduction of laser ablation in air (LAA), where different optical probing techniques are introduced and compared regarding their resolution and obtainable physical quantities. This is followed by an in-depth review of using optical probing techniques to obtain a concise picture of LAL dynamics on timescales ranging from picoseconds up to microseconds.

#### Laser ablation in air

Laser ablation of metals with ultrashort pulses takes place as a cascade of ultrafast processes. After the initial absorption of the laser pulse within the optical penetration depth, electronic thermalization via electron–electron scattering leads to a sharp increase in electron temperatures within a few to tens of femtoseconds [154–155]. Due to the comparatively slow electron–phonon coupling, the heating of the lattice is delayed by about 1 to 20 ps, depending on the material [156–158]. In parallel, strongly temperature-dependent electron transport [159] drives spatial heat flow with typically two distinct regimes of heat diffusion [160]. The theoretical description of these processes is made mainly using the TTM, which describes the energy flows between the electron and phonon subsystems using coupled Fourier equations [20]. This applies to pulse lengths that exceed the electron–electron thermalization time and fall below the electron–phonon interaction time. For typical lattice heating rates of about 1 kK/ps, isochoric heating leads to a pressure increase of several gigapascals, provided the stress confinement condition is met [50,161–163]. Depending on the fluence, these high stresses lead to different ablation mechanisms. Near the ablation threshold, photomechanically initiated spallation occurs, where a thin liquid layer of a few nanometers is ablated by mechanical fracture after the formation of subsurface voids due to extensive tensile stresses [38,49–50,161]. If the temperature exceeds about 90% of the thermodynamic critical temperature, a photothermal phase explosion occurs in which the material decomposes by explosive boiling into a mixture of vapor and liquid droplets [24,164–166]. At even higher fluence values, the ablation plume can be ionized and forms plasma-like states, such as those utilized in pulsed laser deposition [167]. For pulse durations exceeding the stress confinement condition, the ablation process becomes rather fluence-independent and is primarily photothermal [161,168]. The mechanisms of these process dynamics can only be understood fully by a combination of simulations with suitable pump–probe techniques, which provide time-resolved observables. Common pump–probe methods and their results are presented in the following.

#### Summary of common pump–probe methods using laser ablation in air as an example

The dynamics of ultrashort-pulse laser ablation on timescales of femtoseconds to picoseconds exceed the temporal resolution of conventional optical sensors, whose integration times are typically in the range of nanoseconds to milliseconds. As early as 1967, Shelton et al. [169] used an optical ultrashort-pulse pump–probe setup for the first time to investigate the lifetimes of color centers in crystals by means of transmission measurements. The basic principle, a pump pulse and a split, time-delayed probe pulse that scans the transient change in the target using an optical delay line, is still used today [170].

Pump–probe measurements to study the interactions between femtosecond laser pulses and bulk targets, were first presented in 1983 by Shank et al. [171–172] and Eesley [173]. These early works, which were not yet imaging, focused on time-dependent reflectance changes to investigate processes such as the heating of electrons and lattice in copper [173]. In this way, deeper insights could be gained into the TTM introduced by Anisimov in 1974 [20]. In addition, the metallic properties of liquid silicon made it possible to observe phase transitions such as melting [172].

In subsequent years, the non-equilibrium dynamics between electrons and phonons were mainly investigated using pump–probe reflectometry, under induced temperature changes of a few to several thousand kelvins. This research, often in combination with theoretical modeling, focused primarily on metals [174–179]. In addition, ultrashort-pulse laser-induced phase transitions in semiconductors have been investigated [129], and first mechanistic material responses, such as early hydrodynamic motions at lattice heating rates of up to kilokelvins per picoseconds, have been observed [180].

The first imaging method, pump–probe microscopy (PPM), was introduced in 1984 by Downer and coworkers [181]. With a temporal resolution of about 100 fs and imaging on photographic paper, the authors succeeded in imaging the laser-induced melting and evaporation process in silicon both spatially and temporally. In the late 1990s, von der Linde and Sokolowski-Tinten et al. [182] carried out systematic PPM experiments to investigate the dynamics of the ablation process in dielectrics, semiconductors, and metals. Newton rings were observed for the first time during the ablation of metals and silicon, whose temporal extent could be limited to intervals of a few hundreds of picoseconds to a few nanoseconds [24]. However, at that time, these phenomena were not associated with spallation but were attributed to an expanding inhomogeneous phase. It was not until 2006 that Bonse et al. [183], based on work by Zhakhovskiǐ et al. [184], provided an interpretation that corresponds to the current state of the art.

In 2012, Domke et al. [185] published an extended PPM setup that includes an electronically delayed laser alongside an optical delay. This enabled delay times above several nanoseconds up to the second time scale, with a temporal resolution of 0.5 ns. In recent years, the PPM methodology has been widely used to investigate a wide variety of systems during ultrashort-pulse laser ablation. These investigations cover dielectrics and semiconductors [186–187], metals and alloys [160,170,188–190], as well as transparent and metallic thin films [191–194].

The PPM method used for studying laser ablation typically employs an ultrashort pump pulse in the near-IR at wavelengths of about 800 nm (Ti:sapphire laser) or 1030 nm (Yb-doped laser) with a pulse duration in the sub-picosecond to the picosecond range to initiate the ablation process and a time-delayed probe pulse, which is often generated by frequency doubling of a part of the pump pulse.

The temporal resolution of the method is determined by the duration of the probe pulse. For an “ultrafast movie”, a separate experiment is carried out on a pristine target area for each delay time, especially when irreversible changes of the target are involved. Delay times up to the nanosecond scale (corresponding to an optical path of approx. 30 cm for 1 ns) are realized using optical delay lines; for larger delays, electronically synchronized lasers are employed [185]. This flexibility allows for the investigation of dynamics over nine orders of magnitude. The lateral resolution is limited by the probe wavelength and is typically around 0.5 μm for the frequency-doubled NIR probe pulses, while the pump beam diameter can vary from a few micrometers to several hundred micrometers.

Various optical probing techniques have been developed based on the pump–probe principle. These range from PPM and pump–probe reflectometry [193] to pump–probe shadowgraphy (PPS) [195–196], in which transient quantities such as reflectivity, absorption, and transmission are investigated, to more complex methods such as pump–probe ellipsometry (PPE) [197] to determine the complex refractive index and pump–probe interferometry (PPI) [198–199] to measure temporal phase changes, material bulges, and spallation layer velocities. As explained below, some of these techniques are subject to specific limitations regarding the time window accessible for measurements.

**Figure 11:**
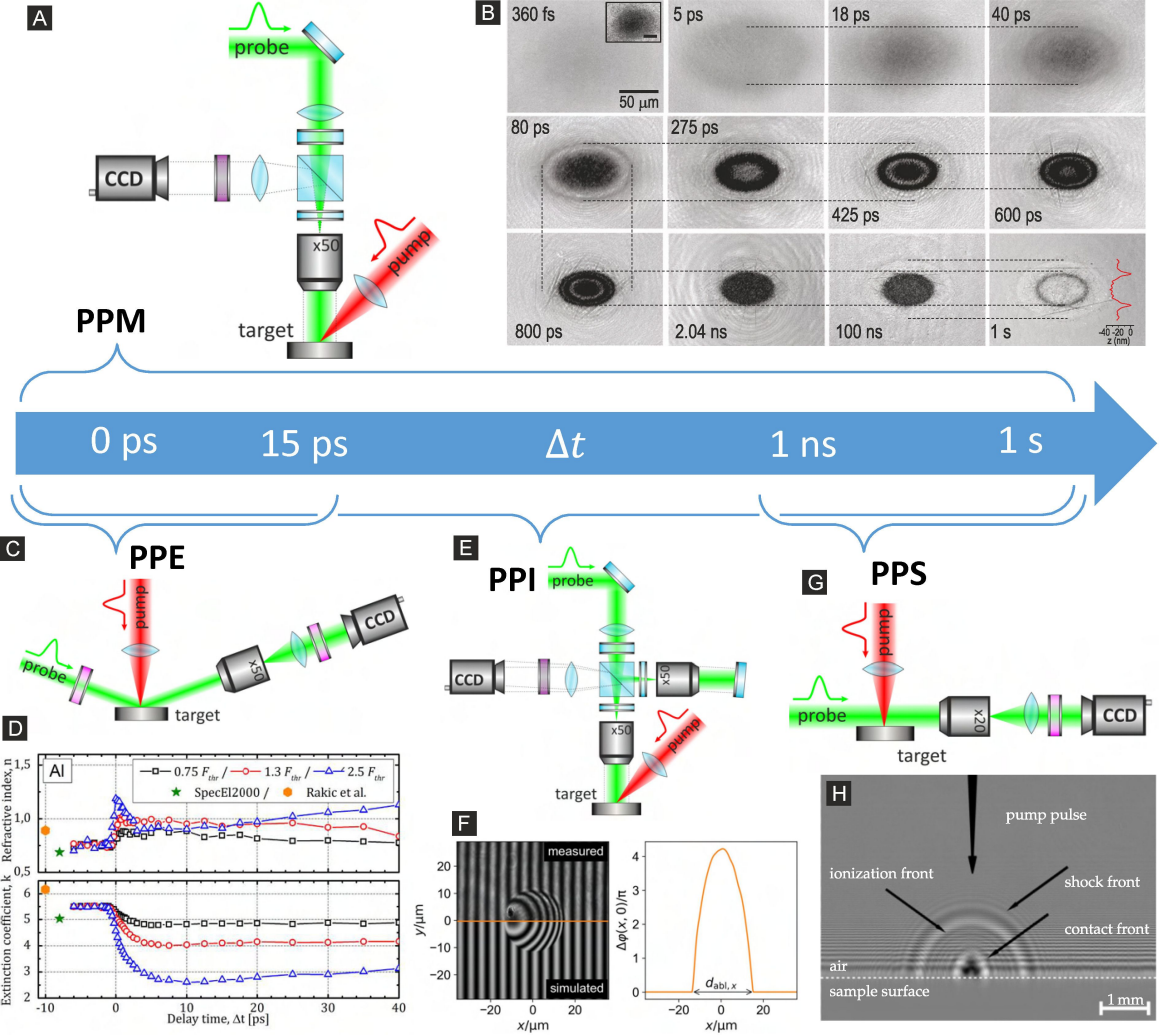
(A) Schematic setup of PPM. The pump pulse (red) is focused on the target at a defined angle, while the time-delayed probe pulse (green) is imaged through polarization and frequency-selective optics and a microscope objective on a CCD camera. (B) Time-resolved PPM measurements on stainless steel at a pump pulse duration of 120 fs and approximately twice the ablation threshold. The time-dependent change in reflection is shown for delay times from 360 fs to 1 s. Noticeable are Newton rings that form because of interference between the spallation layer and the underlying target. (C) Schematic diagram of PPE. A rotating analyzer enables the measurement of the ellipsometric angles used to determine the transient complex refractive index of the target. (D) PPE measurements of the complex refractive index of aluminum, irradiated with 500 fs long laser pulses. (E) Setup of PPI. A Michelson interferometer is integrated into the probe path to generate interference fringes on the CCD detector. The fringes contain the spatial phase information that provides information about surface bulging. (F) Typical PPI measurement. Left: Measured interference fringes caused by the tilt of the reference arm relative to the target arm. Right: Reconstructed phase or surface curvature as a function of position. (G) Schematic diagram of PPS. Shock fronts and ablation plumes can be visualized by lateral illumination with a lateral resolution of about 1 μm. (H) PPS measurement on stainless steel. The plasma ionization front, the shock front in air, and the strongly absorbing ablation plume in the center are shown. Figure 11B was reproduced from [190], (© 2022 The Authors. *Laser & Photonics Reviews* published by Wiley-VCH GmbH, distributed under the terms of the Creative Commons Attribution-NonCommercial 4.0 International License, https://creativecommons.org/licenses/by-nc/4.0/). This content is not subject to CC BY 4.0. Figure 11D was reprinted from [200], *Applied Surface Science*, vol. 511, by J. Winter et al., “Ultrafast Pump-Probe Ellipsometry and Microscopy Reveal the Surface Dynamics of Femtosecond Laser Ablation of Aluminium and Stainless Steel”, article no. 145514, Copyright (2020) with permission from Elsevier. This content is not subject to CC BY 4.0. Figure 11F was reproduced from [199] (© 2024 T. Pflug et al., published by Springer Nature, distributed under the terms of the Creative Commons Attribution 4.0 International License, https://creativecommons.org/licenses/by/4.0). Figure 11H was reprinted with permission from [196] © Optical Society of America. This content is not subject to CC BY 4.0. Figure 11A, C, E and G was inspired by [197].

Figure 11A shows a typical PPM setup for capturing the time-resolved reflectivity of the ablation area over the entire temporal range. The pump pulse (red) is applied to the target at a defined angle, while the time-delayed probe pulse (green) is imaged via polarization and frequency-selective optics and a microscope objective to a camera.

Figure 11B shows exemplary PPM measurements using the example of stainless steel, performed at approximately twice the ablation threshold and a pulse duration of 120 fs [190]. The time-dependent relative reflectance change for different delay times is shown. Already within the first picoseconds, a sharp drop in reflectance, caused by temperature and density changes as well as a melting transition [156,201], is visible, which leads to deep insights into non-equilibrium driven electronic transport and hydrodynamic processes [160,200,202].

For delay times between 250 and 800 ps, characteristic interference patterns, which are called Newton rings and occur due to interference between the spallation layer and the remaining target, appear in the crater center [170,183,190]. These interference patterns determine the spallation layer velocities to about 1 km/s [170,190,200], as well as the spatial extent of the spallation layer [186,190], both depending on the fluence of the pump pulse. In the phase explosion regime, the strongly absorbing and scattering ablation plume causes the almost complete reflectance to drop to zero, typically within 100 ps [163,168,192,203].

In addition, PPM enables the observation of the melting process in semiconductors, which is characterized by the metallic properties of liquid semiconductors and the associated significant increase in reflectance [181,204]. The method also allows for the analysis of the propagation of shock waves in transparent materials [205] or at the air–target interface [195].

PPE proves to be an extremely powerful tool to get access to the absorptance and the reflectance of surfaces [194,197,206–208]. PPE gives feasible values as long as the Fresnel approximation at the surface is valid, which holds as long as structural changes within the optical penetration depth are small [201]. This is typically the case for the delay times up to 50 ps. As early as 1974, PPE was introduced by Auston et al. [206], even before the development of PPM, to investigate the time-dependent generation of free charge carriers in laser-irradiated germanium. Figure 11C shows a more contemporary rotating analyzer PPE setup, based on the work of Rapp and coworkers [197]. For investigations on bulk targets, the measured time-dependent ellipsometric angles are typically calculated into complex refractive indices using the pseudo-dielectric function [209]. PPE thus provides two measured variables for each delay time, the real and imaginary components of the complex refractive index. Figure 11D illustrates an exemplary PPE measurement of the complex refractive index of aluminum, irradiated with a 500 fs laser pulse at various multiples of the ablation threshold [200]. As indicated here, the measurement is limited to a delay time of 40 ps, provided that structural changes, such as spallation or phase explosion, remain within the Fresnel constraints. Such measurements are usually considerably more complex to analyze than PPM data, as they require comprehensive modeling to obtain reliable and quantitative information about the process dynamics, such as electron and lattice temperatures or density [201].

An extension to PPM is PPI [198–199]. With this method, not only the intensity, but also the phase of the probe pulse, can be captured during the ablation process, which enables investigations of surface bulging, for example before the spallation process, with an accuracy in the nanometer range. Technically, the method is realized by integrating a Michelson interferometer into the probe path of a PPM setup, as shown in Figure 11E. This causes interference lines on the detector, which are due to the tilt of the reference arm relative to the target arm (see Figure 11F, left graph) [198]. The modulated lines contain the spatial phase information (Figure 11F, right graph) [199]. These phase shifts can be caused by both time-varying optical properties of the target and optical path differences of the bulging surface. Therefore, the analysis requires accurate modeling and precise temporal boundaries [198]. Combining measurements of the complex refraction index by PPE and optical phase shift in the context of PPI with a validated model could offer new possibilities in the future to resolve density and temperature on an ultrafast timescale.

A further variation of PPM is PPS. This technique has proven to be extremely powerful, particularly in LAL, in creating lateral images of the transmission of ablation plumes, shock dynamics, and bubbles [195–196,210–214]. The side illumination shown in Figure 11G with a lateral resolution of about 1 μm made it possible for the first time to visualize processes in the nanosecond range since shock or spallation fronts expand at speeds in the range of kilometers per second. Figure 11H shows an example of a typical PPS measurement on stainless steel. The plasma ionization front, the shock front in air, and a strongly absorbing ablation plume in the center can be seen [196]. Especially for the analysis of the ablation plume on nanosecond timescales and with respect to its interaction with subsequent pulses of gigahertz or megahertz bursts, PPS proves to be an extremely effective tool [215–217].

Usually, the combination of different experimental techniques leads to a more comprehensive, quantitative understanding of the processes, which can be further matched with simulation data. While the ablation process of metals in air has already been investigated intensively, new effects are emerging for LAL.

#### Optical probing of laser ablation in liquid dynamics

In contrast to LAA, LAL is performed by immersing a target under a liquid layer with the aim of generating ligand-free NPs from virtually any material [1]. For this, most commonly ultrashort pulses with durations from several hundreds of femtoseconds to tens of pisoseconds or short pulses with pulse durations of about 10 ns are used. The liquid layer can either be an organic or an inorganic solvent. While the inorganic solvent water is most commonly used, organic solvents are desirable when processing oxidation-sensitive targets [19]. Furthermore, additives such as NaCl can be added in micromolar concentrations to the solvent to promote electrostatic stabilization of the produced NPs and prevent ripening within the liquid environment [218]. The liquid layer not only acts as the suspension medium for the generated NPs, but it also allows for the control over NP properties [4,19] and facilitates high lattice cooling rates of up to 10 K/ps [33,125,219], promoting the generation of defect-rich NPs [220]. However, the presence of the liquid layer leads to laser energy losses [221] and alters the ablation dynamics on all timescales ranging from picoseconds to milliseconds [222].

In the following section, we will focus on water as an immersion medium and summarize the ablation dynamics induced by laser pulses as deduced by optical probing methods. These dynamics are presented chronologically, starting with the non-linear interaction of the laser pulse with the liquid top layer, followed by the ablation dynamics in the sub-nanosecond and nanosecond/microsecond ranges.

#### Pulse energy losses within the liquid layer

In LAA, the laser pulse energy can be delivered to the sample material without significant energy loss as, even for pulse durations of 120 fs, optical breakdown within air is only observed for very high fluences exceeding 26 J/cm^2^ [223]. These fluences are far above typical values employed in LAA, which lie for an efficient process in the range of several joules per square centimeter, corresponding to a few multiples of the ablation threshold [224]. For LAL, in contrast, the liquid immersion layer causes linear absorption losses. For example, water exhibits an absorption coefficient of 0.144 cm^−1^ [225] at a laser wavelength of 1064 nm, thus resulting in linear absorption losses of several percents for typical water layers of several millimeters thickness.

**Figure 12:**
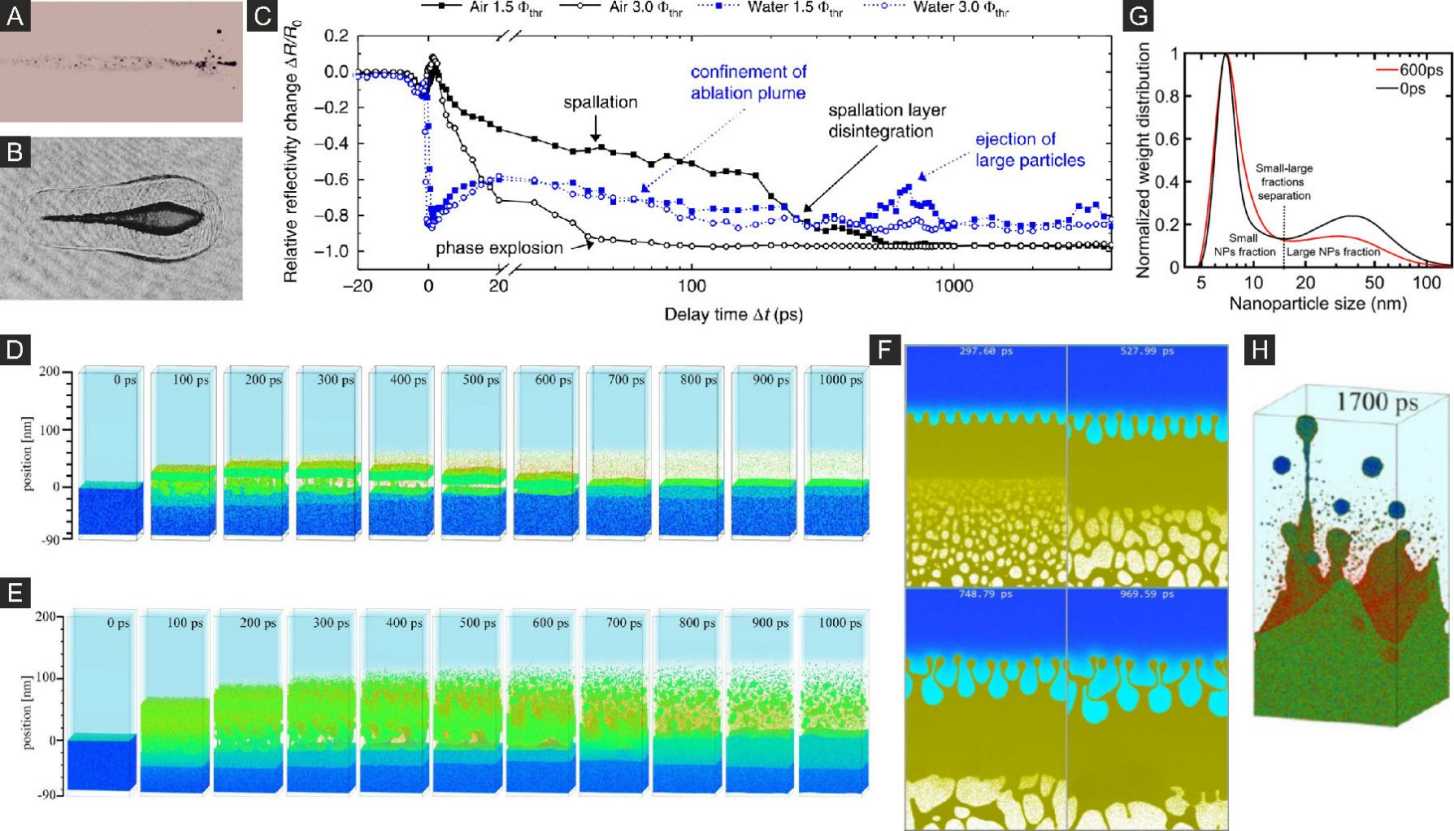
Sub-nanosecond laser ablation dynamics in liquid. (A) Filament formation after irradiation of distilled water with a wavelength of 775 nm, a pulse duration of 150 fs, and a pulse energy of 785 μJ. The laser pulse was incident from the left. (B) Optical breakdown formation after irradiation of distilled water with a wavelength of 1064 nm, a pulse duration of 30 ps, and a pulse energy of 1 mJ. The laser pulse was incident from the left and the image was taken 44 ns after pulse impact. (C) Relative reflectance change Δ*R*/*R*_0_ as a function of the delay time Δ*t* following irradiation of a gold sample immersed in air (black) and water (blue) with a wavelength of 1056 nm, a pulse duration of 3 ps, and peak fluences of 1.5Φ_thr_ (squares) and 3.0Φ_thr_ (circles). Here Φ_thr_ denotes the single-pulse ablation threshold fluence. (D, E) Snapshots of MD simulations of a FeNi target immersed in water irradiated with a 10 ps laser pulse with absorbed peak fluences of 0.08 J/cm^2^ (D) and 0.135 J/cm^2^ (E). (F) Snapshots of MD simulations of a bulk Au target immersed in water irradiated with a 100 fs laser pulse with an absorbed peak fluence of 0.4 J/cm^2^. (G) Normalized NP weight distribution produced by double-pulse ablation of gold in water with a 1064 nm, 10 ps, and 13 J/cm^2^ laser. Two inter-pulse spacings of 0 ps and 600 ps are shown. (H) Snapshots of MD simulations of a bulk Ag target immersed in water irradiated with a 100 fs laser pulse with an absorbed peak fluence of 0.3 J/cm^2^. Figure 12A was adapted from [228] (© 2022 D. C. K. Rao et al., published by Springer Nature, distributed under the terms of the Creative Commons Attribution 4.0 International License, https://creativecommons.org/licenses/by/4.0). Figure 12B was adapted with permission from [236] Copyright 1996, Acoustical Society of America. This content is not subject to CC BY 4.0. Figure 12C was reproduced from [158] (© 2022 M. Spellauge et al., published by Springer Nature, distributed under the terms of the Creative Commons Attribution 4.0 International License, https://creativecommons.org/licenses/by/4.0). Figure 12D and E is from [219] (C. Chen et al., “Atomistic modeling of pulsed laser ablation in liquids: spatially and time-resolved maps of transient nonequilibrium states and channels of nanoparticle formation”, *Applied Physics A*, vol. 129, article no. 288, published by Springer Nature, 2023, reproduced with permission from SNCSC). This content is not subject to CC BY 4.0. Figure 12F is from [237] (N.A. Inogamov et al., “Dynamics of Gold Ablation into Water”, *Journal of Experimental and Theoretical Physics*, vol. 127, pages 79–106, published by Springer Nature, 2018, adapted with permission from SNCSC). This content is not subject to CC BY 4.0. Figure 12G was used with permission from [238] (“Double-pulse laser ablation in liquids: nanoparticle bimodality reduction by sub-nanosecond interpulse delay optimization”, by C. Doñate-Buendia et al., *J. Phys. D: Appl. Phys.*, vol. 56, issue 10, article no. 104001, published on 22 February 2023; https://doi.org/10.1088/1361-6463/acbaaa); © 2023 IOP Publishing Ltd; permission conveyed through Copyright Clearance Center, Inc. All rights reserved. This content is not subject to CC BY 4.0. Figure 12H was adapted from [239], C.-Y. Shih et al., “Generation of Subsurface Voids, Incubation Effect, and Formation of Nanoparticles in Short Pulse Laser Interactions with Bulk Metal Targets in Liquid: Molecular Dynamics Study”, *J. Phys. Chem. C*, © 2017 American Chemical Society, distributed under the ACS AuthorChoice/Editors’ Choice via Creative Commons CC-BY Usage Agreement, https://pubs.acs.org/page/policy/authorchoice_ccby_termsofuse.html. This content is not subject to CC BY 4.0.

Furthermore, the pulse intensities can create non-linear effects including filamentation [226] (see Figure 12A) and optical breakdown [227] (see Figure 12B). The lack of inversion symmetry in liquids gives rise to a third-order non-linear susceptibility χ*^(^*^3^*^)^*, facilitating degenerate four-wave mixing, resulting in self-focusing and self-phase modulation [226]. For femtosecond pulses, self-focusing is typically observed in liquids for peak powers of approximately 1 MW [228]. Importantly, it is the beam power and not the intensity that determines the onset of self-focusing [226,229]. Whole-beam self-focusing leads to a distortion of the beam profile and, exceeding a certain threshold, to optical breakdown. Small-scale distortions in the spatial beam profile give rise to the development of filaments [230]. It was reported that this filamentation process can result in an absorption of up to 46% [231]. In the case of optical breakdown, a plasma with an electron density of typically 10^21^ cm^−3^ is generated within the liquid once threshold intensities in the range of several 10^12^ W/cm^2^ for femtosecond pulses [232], several 10^11^ W/cm^2^ for picosecond pulses [233], and several 10^10^ W/cm^2^ for nanosecond pulses [233] are exceeded. The plasma generation within the liquid is driven by either multiphoton absorption (femtosecond pulse duration) or cascade ionization (nanosecond pulse duration) or a combination of both processes (pisosecond pulse duration) [234]. All lead to vaporization of the liquid followed by shockwave and cavitation bubble (CB) formation [227] (see Figure 12B). Reported absorption losses due to optical breakdown within the liquid range from up to 30% at six times the breakdown threshold for a 10 ps pulse, to 70% at 60 times the breakdown threshold for few-picosecond pulses [235]. This demonstrates that optical breakdown presents a significant loss mechanism in LAL.

A way to circumvent this is simultaneous spatial and temporal focusing, enabling to double LAL productivity compared to conventional optical systems [231]. Finally, alongside others, self-phase modulation leads to a broadening of the laser pulse spectrum, which may be utilized for supercontinuum generation [240]. These processes demonstrate that the liquid layer already limits the available pulse energy before the laser pulse impacts the target material. Once the laser pulse energy reaches the target surface, dynamics are triggered that differ significantly between LAL and LAA as outlined in the next section.

#### Sub-nanosecond laser ablation in liquid dynamics

Once the laser pulse reaches the targets surface, the pulse energy is absorbed by the electronic subsystem [241]. In the case of LAA, the absorption depends strongly on fluence and pulse duration as the leading edge of the laser pulse heats the target sufficiently to alter its optical properties, which in turn influences the absorptance for the trailing edge of the laser pulse [242]. This phenomenon known as self-reflection may alter the absorptance of the target by up to one order of magnitude compared to the low-fluence absorptance value [242–243]. Following absorption of the laser pulse energy by the electron subsystem, the energy is transferred to the lattice by electron–phonon coupling [241] on a timescale determined by the electron–phonon relaxation time, which is of the order of 1 ps for strongly coupling targets such as stainless steel and 10 ps for weakly coupling targets such as Cu [200]. Up until this point, the laser energy absorption and temperature equilibration are comparable for LAA and LAL [222].

Recent experimental works on LAL highlighted that the influence of the liquid overlayer is already detectable a few picoseconds before and after delay time zero. As shown in Figure 12C, the reflectance for ablation of Au for LAA with a 3 ps pulse drops and rises within a range of relative reflectance change of Δ*R*/*R*_0_ ≈ −10% to Δ*R*/*R*_0_ ≈ +10%, whereas for ablation in water a non-linear decrease to about Δ*R*/*R*_0_ ≈ −85% is observed faster than the laser pulse duration [158]. Similar reflectance dynamics were also observed for the ablation of Fe in water with a pulse duration of 500 fs [189]. In the case of LAA, the drop and rise in reflectance shortly before and after pulse impact is attributed to increasing temperature and decreasing density [200–201,244]. For LAL, in contrast, detailed investigation of the fast reflectance decrease by PPM [158,189] showed that thermionic electron emission from the hot target generates a sufficient amount of free electrons within the liquid to induce an optical breakdown closely above the target’s surface. While this effect could be clearly identified by the time-resolved optical experiments, its influence on key LAL outcomes such as ablation efficiency and particle size distribution is still unknown.

These early phenomena are followed by the expansion and the ablation of material. Experimental and computational works have identified photomechanical and photothermal ablation mechanisms for LAA [161,245]. For peak fluences ranging from the ablation threshold Φ_thr_ to approximately 2Φ_thr_ photomechanical spallation is the driving ablation mechanism [244]. Here, isochoric heating of the target within the regime of stress confinement [50,161] leads to the generation of compressive shockwaves that reflect at the free surface of the heated volume, thereby producing tensile stresses of the order of 10 GPa [161]. These stresses are sufficiently strong to overcome the tensile strength of the material and cause fracture and ejection of a molten layer [246]. In addition to spallation, such laser-driven shockwaves have been shown to induce phase transitions, for example, the transformation of graphite into diamond under nanosecond shock compression at pressures exceeding 50 GPa [247]. A related industrial application is laser shock peening, which employs shockwaves generated under a liquid confinement layer to induce plastic deformation and residual compressive stresses that enhance the fatigue resistance and mechanical durability of metal surfaces without causing ablation [248]. In the relative reflectance change in Figure 12C (black dots), the spallation process specifically manifests at a peak fluence of 1.5Φ_thr_ as a slowly decaying non-zero reflectance between 10 and 200 ps due to finite reflectance of the propagating spallation layer [158]. The spallation front can be monitored by time-resolved optical probing with the appearance of Newton rings [24]. These rings arise because of interference of the probe pulse between the upward propagating spallation layer and the underlying surface [249]. The deduced spallation layer velocities are strongly fluence-dependent and range from hundreds of meters per second closely above the ablation threshold fluence [190] to nearly 2 km/s at 2.5Φ_thr_ [200]. Notably, the Newton rings can only be observed when the ejected spallation layer is thin enough to be partially transmissive for the employed probe wavelength, which is typically the case when the spallation layer thickness is comparable to the optical penetration depth [170]. When applying peak fluences larger than 2Φ_thr_, the laser pulse heats the targets surface to temperatures close to the critical temperature, and photothermal phase explosion is initiated [250]. Here, the material decomposes into liquid droplets and vapor [161]. In the PPM signal in Figure 12C (black open circles), this mechanism is observed by the drop of reflectance to 0 (Δ*R*/*R*_0_ = −1) after 100 ps due to scattering and multiple absorption of the probe pulse in the droplet/vapor mixture [158]. As this short overview shows, the ablation mechanisms of spallation and phase explosion were extensively studied for LAA.

Similar ablation mechanisms are also expected to occur for LAL [158,219,222,239]. However, in the case of LAL the material expands against the liquid layer, which in turn exerts recoil forces on the ablated material and decelerates it [237,239]. This can be indirectly monitored by PPM as shown in Figure 12C (blue circles). Here for LAL the transient reflectance in the delay time range of 20 to 200 ps exhibits a non-zero value independent of the applied peak fluence, implying that a Fresnel-like boundary is present between the liquid layer and the emerging ablation plume [158,189]. This shows the confinement of a liquid ablated material layer also at the higher fluence (open blue circles, Figure 12C) in the phase explosion regime by the surrounding water since a drop of the reflectance to −1 (zero reflectance) would be expected without the presence of the liquid layer [158,245,251]. As shown in Figure 12D, the confinement of the ablation plume is predicted by MD simulations for ablation of Fe_0.5_Ni_0.5_ within the spallation regime [219]. Here, the ejected spallation layer is confined by the liquid and eventually redeposited to the target surface, while only a small amount of ablated material is converted into NPs and clusters. Notably, the timescale for the confinement from about 50 to 500 ps agrees well between computational predictions (Figure 12D) and experimental observations (Figure 12C). Furthermore, when moving to the phase explosion regime (Figure 12E) the MD simulations again predict the confinement of the ablation plume, which is validated with the experimental observations. However, in this case, a larger portion of the material is ejected into the surrounding liquid, but a rather sharp interface between the ablation plume and liquid is still present, explaining the observation of non-zero reflectance observed by PPM within the phase explosion regime (Figure 12C, blue open circles).

At this point, it should be noted that the MD simulations predict that approximately 80% of the ablated materials is redeposited onto the target surface [219]. This highlights that the alteration of the ablation dynamics when moving from air to liquid and the associated redeposition results in a decreased ablation efficiency. However, up until now, the amount of redeposited material has not been quantified experimentally. For LAA, it was observed that the reflectance drops between 200 ps and 1 ns (see Figure 12C, black full circles) within the spallation regime. This reflectance drop was interpreted as a disintegration of the spallation layer into smaller liquid droplets, which then causes complete extinction of the incident probe pulse and thus the observation of zero reflectance [158]. For LAL, the disintegration of the spallation layer into larger particles is expected to occur on a similar timescale as deduced from MD simulations (see Figure 12D and Figure 12E). Optical probing of the LAL dynamics within the time interval reveals a peculiar reflectance increase around a delay time of 600 ps (see Figure 12C). This rise was attributed to the disintegration of the spallation layer into larger NPs, which could cause an increased reflectance due to the plasmon resonance. In fact, the optical probing at 528 nm lies very close to the plasmon resonance peak of Au NPs located around 530 nm for sizes larger than approximately 10 nm [252]. Thus, optical probing of the LAL process allows one to identify the generation of larger NPs after 600 ps. This is in accordance with computational predictions, where MD simulations of Au irradiated in water show that the ejected spallation layer disintegrates into larger particles on a similar timescale (see Figure 12F) [237].

Further optical experiments have been conducted to underpin the formation of larger NPs on a sub-nanosecond timescale. For this, double-pulse laser ablation of Au in water was performed with two identical pulses that were separated by an inter-pulse delay ranging between 0 ps and 1.2 ns [238]. Instead of transient reflectance, the obtained NP size distribution was monitored dependent on the inter-pulse delay time (see Figure 12G). For an inter-pulse delay of 0 ps (black line), which corresponds to single-pulse ablation, the particle size distribution showed a strong bimodality typical for ultrashort LAL of metal targets [253]. This bimodality may well be a result of two channels of NP generation as predicted by simulations (see Figure 12H) [239]. Small primary NPs with diameters of several nanometers are generated by condensation of the metal vapor (small NPs in Figure 12H), whereas large NPs with diameters of several tens of nanometers are generated by disintegration of the ejected spallation layer due to thermodynamic instabilities (large NP in Figure 12H) [43,254]. When the inter-pulse delay in the double-pule experiments was increased, it was observed that the amount of large particles decreased gradually until a minimum amount was measured at an inter-pulse delay of 600 ps (see Figure 12G, red line). A further increase in the inter-pulse delay resulted again in a higher bimodality. The interaction of a second pulse with the ablation plume again demonstrates that a significant amount of large particles is generated within the time interval around 600 ps in accordance with time-resolved probing of the reflectance signal (see Figure 12C, open blue circles) and computational predictions (see Figure 12F). As the efficiency of the double-pulse ablation process exhibited a minimum at an inter-pulse delay of 600 ps, it was speculated that a fraction of the absorbed pulse energy was used to fragment the larger 30 nm particles into smaller 7 nm particles, similar to LFL of laser-generated NPs [12]. Thus, the double-pulse experiments not only help in assessing the timescales for secondary NP formation but also represent a promising method for obtaining monomodal NP size distributions in a single process step [238]. However, further understanding of the downsizing mechanisms in double-pulse LAL is needed to fully utilize the method and obtain monomodal NP size distributions.

In conclusion, the presence of the liquid overlayer significantly alters the sub-nanosecond dynamics of LAL compared to LAA, ranging from thermionic electron emission-mediated optical breakdown over confinement of liquid propagating ablated material and its redeposition to the generation of NPs. These processes determine both the process efficiency as well as the final NP size distribution.

#### Nanosecond, microsecond, and millisecond laser ablation dynamics in liquid

**Figure 13:**
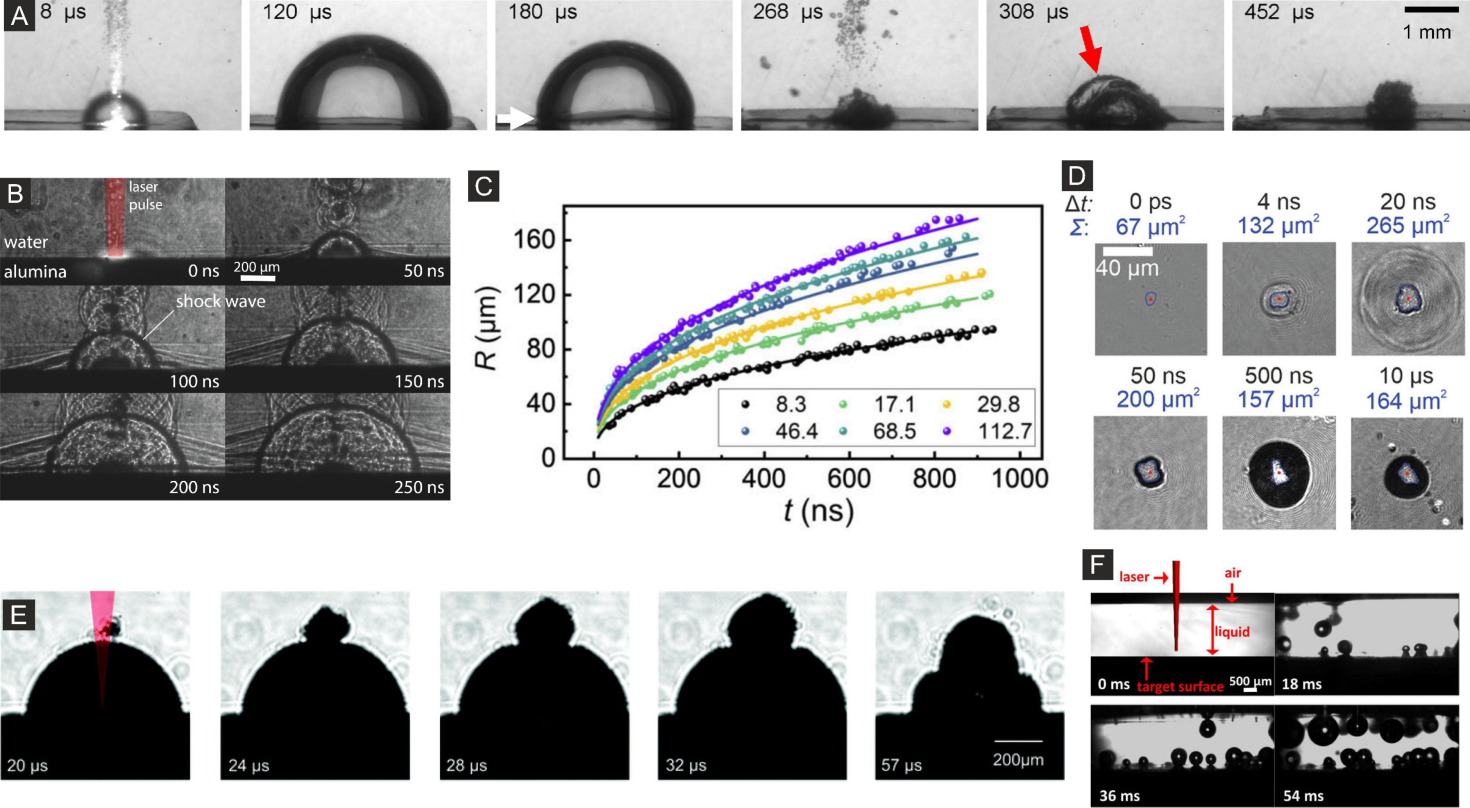
Nanosecond, microsecond and millisecond laser ablation dynamics in liquid. (A) Shadowgraphs with a tilted view depicting the microsecond ablation dynamics of a silver target immersed in water irradiated with a 7 ns, 1064 nm laser pulse with an energy of 16 mJ. The images were generated by using a cw diode laser together with a gated CCD camera. (B) Shadowgraphs depicting the nanosecond ablation dynamics of an aluminum target immersed in water after irradiation with a 5 ns, 355 nm laser with a pulse energy of 60 mJ. (C) Transient evolution of CB radius *R* after irradiation of a titanium sample immersed in water with a 20 ns and 1064 nm laser pulse. Probing was performed by a nanosecond Nd:YAG laser. The evolution of *R* is depicted for different peak fluences given in the legend in units of J/cm^2^. (D) PPM images depicted the entire laser fragmentation dynamics of single IrO_2_ microparticles subjected to single 10 ps, 1040 nm laser pulses with a peak fluence of 0.18 J/cm^2^. (E) Shadowgraphs depicting the interaction of a second laser pulse with the emerging CB. The second laser pulse impacts the bubble 20 μs after generation by the first laser pulse and exhibits a pulse duration of 10 ps, a wavelength of 1064 nm and a peak fluence of 3.4 J/cm^2^. The target is Au immersed in water. (F) Transient development of persistent microbubbles after irradiation of an Au target immersed in water with a pulse duration of 8 ns, a wavelength of 1064 nm and fluence of 27 J/cm^2^. Figure 13A was adapted from [255], *Journal of Colloid and Interface Science*, vol. 489, by S. Reich et al., “Pulsed laser ablation in liquids: Impact of the bubble dynamics on particle formation”, pages 106–113, Copyright (2017), with permission from Elsevier. This content is not subject to CC BY 4.0. Figure 13B was reprinted from [195], *Applied Surface Science*, vol. 574, by A. Chemin et al., “Investigation of the Blast Pressure Following Laser Ablation at a Solid–Fluid Interface Using Shock Waves Dynamics in Air and in Water”, article no. 151592, Copyright (2022), with permission from Elsevier. This content is not subject to CC BY 4.0. Figure 13C was reprinted with permission from [259] © Optical Society of America. This content is not subject to CC BY 4.0. Figure 13D was adapted from [260] (© 2023 The Authors. *Small* published by Wiley-VCH GmbH, distributed under the terms of the Creative Commons Attribution-NonCommercial 4.0 International License, https://creativecommons.org/licenses/by-nc/4.0/). This content is not subject to CC BY 4.0. Figure 13E was reproduced from [43] (“Two Mechanisms of Nanoparticle Generation in Picosecond Laser Ablation in Liquids: The Origin of the Bimodal Size Distribution”, © 2018 C.-Y. Shin et al., published by RSC, distributed under the terms of the Creative Commons Attribution 3.0 Unported License, https://creativecommons.org/licenses/by/3.0/). Figure 13F was adapted with permission of The Royal Society of Chemistry, from [261] (“How persistent microbubbles shield nanoparticle productivity in laser synthesis of colloids – quantification of their volume, dwell dynamics, and gas composition” by M.-R. Kalus et al., *Phys. Chem. Chem. Phys.*, vol. 19, issue 10, © 2017); permission conveyed through Copyright Clearance Center, Inc. This content is not subject to CC BY 4.0.

The sub-nanosecond LAL dynamics are mainly governed by the ablation of a liquid material layer followed by distinct NP generation channels. The dynamical processes following this are characterized by shockwave emission as well as CB expansion, rebound, and collapse, release of the NPs into the surrounding liquid [158,222]. As shown in Figure 13A–C, these dynamical processes span several orders of magnitude in time; they start on a nanosecond, continue on a microsecond, and are approximately finished on a millisecond timescale [158,222,255]. The shadowgraphy images depicted in Figure 13A were generated by an incoherent illumination of the ablation scene. However, incoherent illumination is typically employing flashlamps as light sources, which display a limited temporal resolution in the range from several tens of nanoseconds [256] to several microseconds [255]. This hinders probing of nanosecond ablation dynamics, such as shock waves being emitted with speeds of up to 12 km/s [257] and the formation of the CB [222]. For probing these phenomena, sub-nanosecond temporal resolution is required. This is typically done with coherent laser sources, which, however, suffer from coherent imaging artifacts, such as ringing and speckles [258]. Furthermore, high numerical aperture illumination is required for the observation of the ablation plume inside the CB [258]. Currently, there are efforts in the development of incoherent illumination sources with short pulse durations, which utilize the amplified spontaneous emission from a rhodamine dye with a temporal resolution of 100 ps [258]. However, the literature on applying these novel sources for LAL is currently extremely sparse. Thus, we will focus next on the nanosecond CB and shockwave dynamics probed with coherent light sources. The preceding shockwave, which generates a low-pressure region in the liquid, plays a key role in initiating this cavitation process by rapidly reducing the local pressure below the vaporization threshold, thereby enabling bubble nucleation and early CB expansion [259].

The CB is initially generated as a flat vapor layer a few 10 ps after pulse impact due to heating of the surrounding liquid by the hot ablation front from target [239]. The generated vapor then rapidly expands, giving rise to the formation of a round CB, filled with the ablation plume containing gaseous as well as liquid species as well as evaporated solvent molecules [222]. However, optical probing of the CB on these early timescales is extremely challenging, as computational methods predict that the CB has expanded by just a couple of 100 nm at a delay time of 1 ns after pulse impact [239]. Nonetheless, at higher fluences and delay times of 1 to 2 ns, the CB detaches from the ablation plume and becomes sufficiently large to be optically detected [259]. As it acts as a focusing lens, it can also be readily detected in PPM experiments [158]. This result has high application relevance as it marks the time point from which subsequent laser pulses significantly interact with the emerging CB [262]. The CB generation time of about 1 ns not only determines a minimal time interval in which the ablated material may still be directly influenced by subsequent pulses, but also explains the high ablation efficiency when employing pulse durations of about 1 ns as the laser pulse does not experience bubble shielding on this timescale [262]. Moving on to longer timescales of several nanoseconds, the CB and shockwave expansion can be clearly detected in PPM [158,262] and PPS [195,259] experiments (see Figure 13B). From these images, the transient evolution of the CB radius may be extracted (see Figure 13C). Modeling based on the Rayleigh–Plesset equation [257,259,263–264] may then be used to obtain quantitative data from the CB evolution, assuming spherical symmetry and incompressibility [265]. For higher accuracy, especially under strong driving pressures, compressibility can be included via the Gilmore [266] or Keller–Miksis [267] models. In cases involving non-spherical collapse or boundary effects, numerical solutions of the Navier–Stokes equation become necessary [268]. Regarding the conversion of absorbed laser energy into CB energy, values strongly depend on pulse duration and irradiation conditions. For nanosecond pulses, conversion efficiencies of approximately 10% have been reported [259], and values up to 33.5% at 532 nm and 53% at 1064 nm have been observed at ten times the optical breakdown threshold fluence [269]. In contrast, femtosecond-induced optical breakdown results in significantly lower conversion efficiencies, with reported values of only 0.033% of the absorbed pulse energy contributing to bubble formation [232]. Quantitative estimations not only help in understanding under which conditions the NPs grow within the CB, but also aid the understanding of the mechanism for laser fragmentation, for example, shown for the LFL of single IrO_2_ microparticles [260]. Here the quantitative analysis of the shockwave (see Figure 13D, 4 ns and 20 ns) and CB (see Figure 13D, 500 ns) dynamics allows for the determination of the pressure inside the microparticle, which exceeds the tensile strength of the particle, highlighting a predominate photomechanical mechanism for the fragmentation [260].

Moving on from the nanosecond to the microsecond regime, the CB further expands until reaching its maximum radius after several tens of microseconds [270–272] to hundreds of microseconds [273–274]. Within this regime, irradiation of the CB by a second pulse was studied (see Figure 13E) [43], which indicated that a significant portion of the incident laser pulse energy is absorbed at the interface of the CB and the surrounding liquid, which highlights the strong shielding effect of the CB [275]. Furthermore, the generation of small satellite bubbles above the CB (see Figure 13E), is associated with the second laser pulse being absorbed by NPs that are present above the CB boundary and thus confirms the jetting of large secondary particles into the surrounding liquid as predicted by MD simulations [43]. Furthermore, small-angle X-ray probing experiments revealed that particle growth and agglomeration happen within the CB, and NP size quenching by adding electrostatic stabilizers to the liquid is already in effect inside the CB [276].

Following maximum extension, the CB starts to collapse on a timescale of hundreds of microseconds [255,270,273–274]. The collapse emits a secondary shockwave, which can transiently raise pressure and temperature in the liquid and may even trigger phase transitions [277]. After CB collapse, a large amount of NPs previously trapped inside the CB is released into the liquid [255,278]. This highlights that subsequent pulses at the same position can only be transmitted to the sample surface several 100 μs after the preceding pulse. For this reason, fast polygon scanners enabling scanning speeds of up to 500 m/s are employed during LAL, to spatially bypass the CB, yielding record NP productivity in the grams per hour range when a laser repetition rate of 10 MHz was employed [275]. Similarly, spatial beam splitting was performed to split the pulse energy into several spatially distributed sub-beams. With several beams the available high laser pulse energy is fully leveraged at reduced repetition rates, while individual laser pulses bypass the CB [279].

Although the ablation dynamics have reached essentially a final state several hundreds of microseconds after pulse impact, persistent microbubbles [255] may remain attached to the targets surface for several tens of milliseconds (see Figure 13F) [261]. For LAL performed in water, persistent microbubbles can shield up to 25% of the incoming laser radiation [261]. This necessitates the use of flow-through LAL reactors, where generated microbubbles together with NPs are consistently removed in order to guarantee high productivity [280–281].

## Conclusion and Outlook

Apparently straightforward laser processes like LAL, LFL, or LML are easily executed using a pulsed laser and a beaker with the materials, but involve a cascade of subsequent energetic and structural relaxations that are difficult to disentangle. The endeavor to master and optimize these laser synthesis processes for specific applications like biophotonics, catalysis, or nanoelectronics requires a stringent and causal identification of the structure formation steps, which is not achievable by the analysis of the final products in a batch process alone.

True understanding has to take advantage of state-of-the-art diagnostics with high temporal and spatial resolution. This review has showcased that by applying structural methods, such as UED and time-resolved SAXS and WAXS to ultimately image single nanoscale objects by coherent imaging, as well as advanced ultrafast optical imaging, a wealth of observations can be made and directly compared to theory and simulation. WAXS and UED address atomic motion in complex systems allowing one to disentangle excited states of irradiated NPs and their interaction with the environment, typically a fluid phase. SAXS and CDI show shape evolution in fragmented or growing NPs and even visualize femtosecond dynamics of single objects in a single excitation cycle at near-atomic resolution. These methods, however, are costly and can only be realized at a few accelerator facilities around the world. Therefore, XFEL and synchrotron studies are restricted to analyzing fundamental processes, which enable transferring the knowledge to applied systems. Yet, access to these facilities is greatly facilitated by charge-free calls for experiments that are granted on a pure merit basis. Optical probing is a powerful tool for the investigation of LAL dynamics as it allows one to probe the entire ablation process with unprecedented temporal resolution. This not only allows one to experimentally validate state of the art LAL models. It also paves the way to achieve record LAL productivity by identifying key limiting factors and properly addressing mitigation strategies to overcome the limits. Based on the already impressive findings enabled by optical probing of the LAL dynamics, future research directions might include employing the PPI technique to LAL to directly probe the propagating ablated material front in the liquid confinement and follow its redeposition in the picosecond time range. The PPI method is especially suited for this task as it allows one to probe material expansion with unprecedented resolution down to a couple of nanometers [198]. Furthermore, spectroscopic pump–probe techniques may present a unique opportunity to study LAL by investigating the spectral signature in a time-resolved manner. This might allow us to precisely study NP generation pathways by spectrally monitoring the onset of NP SPR.

## Data Availability

Data sharing is not applicable as no new data was generated or analyzed in this study.
